# Bivariate extreme value analysis of extreme temperature and mortality in Canada, 2000-2020

**DOI:** 10.1186/s12889-024-18785-3

**Published:** 2024-05-18

**Authors:** Yuqing Zhang, Kai Wang, Junjie Ren, Yixuan Liu, Fei Ma, Tenglong Li, Ying Chen, Chengxiu Ling

**Affiliations:** 1 Academy of Pharmacy, Xi’an Jiaotong-Liverpool University, Ren’ai Road 111, Suzhou, 215123 China; 2 Zhejiang Lab, Kechuang Avenue, Hangzhou, 311121 China; 3 Department of Mathematical Sciences, University of Liverpool, Liverpool, L693BX UK; 4 Department of Financial and Actuarial Mathematics, Xi’an Jiaotong-Liverpool University, Ren’ai Road 111, Suzhou, 215123 China; 5 Yale School of Public Health, Yale University, 60 College Street, New Haven, 06520-0834 CT USA; 6 Department of Applied Mathematics, Xi’an Jiaotong-Liverpool University, Ren’ai Road 111, Suzhou, 215123 China

**Keywords:** Extremal dependence, Bivariate peaks-over-threshold, Extreme temperature and mortality, ARIMA modeling

## Abstract

Climate change increases the risk of illness through rising temperature, severe precipitation and worst air pollution. This paper investigates how monthly excess mortality rate is associated with the increasing frequency and severity of extreme temperature in Canada during 2000-2020. The extreme associations were compared among four age groups across five sub-blocks of Canada based on the datasets of monthly T90 and T10, the two most representative indices of severe weather monitoring measures developed by the actuarial associations in Canada and US. We utilize a combined seasonal Auto-regressive Integrated Moving Average (ARIMA) and bivariate Peaks-Over-Threshold (POT) method to investigate the extreme association via the extreme tail index $\chi$ and Pickands dependence function plots. It turns out that it is likely (more than 10%) to occur with excess mortality if there are unusual low temperature with extreme intensity (all $\chi >0.1$ except Northeast Atlantic (NEA), Northern Plains (NPL) and Northwest Pacific (NWP) for age group 0-44), while extreme frequent high temperature seems not to affect health significantly (all $\chi \le 0.001$ except NWP). Particular attention should be paid to NWP and Central Arctic (CAR) since population health therein is highly associated with both extreme frequent high and low temperatures (both $\chi =0.3182$ for all age groups). The revealed extreme dependence is expected to help stakeholders avoid significant ramifications with targeted health protection strategies from unexpected consequences of extreme weather events. The novel extremal dependence methodology is promisingly applied in further studies of the interplay between extreme meteorological exposures, social-economic factors and health outcomes.

**Supplementary Information:**

The online version contains supplementary material available at 10.1186/s12889-024-18785-3.

## Introduction

Climate change has become a serious threat to the global economy, public health and social development [1]. The World Meteorological Organization [2] reports that disasters attributed to weather, climate and water-related hazards from 1970 to 2019 resulted in over 2 million deaths and $3.64 trillion in losses (i.e., accounting for 50% of records, 45% of related deaths and 74% of related economic losses among all geophysical, meteorological, climatological, hydrological, biological, extra-terrestrial and technological disasters). The number of disasters increased five-fold, and economic losses increased seven-fold over the span of 50 years. Climate change is expected to cause approximately 250,000 deaths per year due to climate-sensitive diseases between 2030 and 2050 [2]. Extreme temperature events (ETEs), particularly those associated with heat, have become more frequent, more intense, and longer lasting [3].

Recent studies reported the frequency, intensity and duration of ETEs influence on mortality, which makes it a pressing public health concern [4, 5]. In particular, large excess mortality has been observed due to extreme high and low temperatures [6]. For example, over 700 people lost their lives in the 1995 Chicago heat wave within one week, while more than 72,000 people died in the 2003 Europe heat wave. In Canada, 619 people in British Columbia died due to the heat during the 2021 heat dome, and 106 deaths died in 2010 heatwave in Quebec. The majority of the deaths occurrhhed in Montreal and were among individuals aged 65 and older. In 2019, a severe cold wave hit the Midwestern United States and Eastern Canada, killing at least 22 people.

Most research focused on the health outcomes resulting from extreme (high or low) temperatures, while little is known about the impact of the exceptionally high frequency of temperature events that are harmful to human life. This paper investigates the influence of extremely frequent adverse temperature events, i.e., frequent heat waves and cold snaps, on excess mortality. This is also motivated by the fact that, the frequency and intensity of heat extreme generally increase [7], while neglecting the extreme temperature changes might underestimate the potential for very serious mortality situations. As shown by [8], both regional and age-group difference existed in the extreme association between frequent exposure to cold/hot temperature and excess death in United States. Therefore, our study aims to identify these differences in Canada, assisting in the government to better allocate healthcare sources to vulnerable population.

In this study, we focus on the association between hazardous-temperature frequency and health consequences. The temperature-mortality relationship was identified by many studies at different temporal and spatial scales, including [9] at annual continent-level worldwide, [10] at small area geographical scale, and [11] even at individual scale. Statistical methods, including time-series analyses and case-crossover designs with or without lagged effects, are commonly used in the aforementioned contributions [12, 13]. In this context, we consider the tolerance to relatively extreme temperatures and focus on the regions with similar temperature patterns [14]. Noting further that, the excessive health crashes (fatalities) are likely to observe when the cumulative effects of unusual temperature over a period of prolonged duration exceed a critical value, we will principally employ extreme value theory (EVT) for quantifying the excess temperature-mortality association [15, 16]. As EVT can provide a reasonable extrapolation from observed states to unobserved states with main focus on the tail of a distribution, it is widely used for modelling and measuring extreme events in hydrology, environment, finance [14, 15, 17], and recently applied in public health settings, such as mortality and morbidity peaks [18, 19], and infectious disease mortality rate [20].

In this paper, the bivariate POT method, as a new research hot-spot of the estimation of extreme values, will be applied since it can fully exploit the extreme property of the observed data [17]. The study of multivariate extremes consists of two steps: the marginal analysis and the dependence study, which is commonly supposed to be marginally invariant. Generally, one may employ marginal tail information to transform the marginal distribution to a common scale, e.g., unit Fréchet distribution. In this paper, we will utilize seasonal Auto-regressive Integrated Moving Average (ARIMA) models to adjust possible trends and seasonality in non-stationary time-series data, and then transform the marginal residuals (excess extreme temperature and excess mortality) into unit Fréchet distribution by generalized Pareto (GP) distributions. Note that the application of the POT approach involves some complexities, and one of the most important issues is the threshold selection since it must balance the trade-off between variance and bias. Several approaches have been developed to determine a proper threshold, and we refer to [21, 22] for a comprehensive review.

The bivariate POT method applied here for the joint tail distribution of extreme temperature and excess mortality has extensive and powerful applications when the linear relationship fails to apply, especially when the extreme phenomenon becomes interesting. Although there were some studies applying POT models to evaluate independent ETEs, few studies considered the bivariate POT method to examine the extreme influence of both extreme low and high temperature frequency on population health except the latest study [8, 14, 23]. Therefore, our results have important implications for comprehensive risk management and mitigation in the healthcare systems, public authorities and environmental agencies, with the aim to help reduce the adverse effects of extreme weather events and improve health protection.

The remaining paper is organized as follows. “Data description” section presents a comprehensive description of the data and its exploratory analysis. “Methodology” section provides a review of the seasonal ARIMA model and the fundamental concepts in EVT, with an emphasis on the bivariate case. “Results” section presents the main results, followed by an extensional discussion of the results in “Discussion” section. Finally, “Conclusion” section concludes this paper.

## Data description

This research was conducted based on a recently developed meteorological time series for severe weather - the Actuaries Climate Index or ACI^1^ (developed by the actuarial associations in Canada and the US), and relevant mortality in Canada over the period 2000–2020. The ACI is a useful severe weather monitoring method to measure and monitor the frequency of severe weather and the extent of sea level rise in the United States and Canada. We chose two important components of the ACI: monthly T90 and monthly T10. Monthly T90 (T10) tracks the change in the frequency of temperature above the 90th percentile (below the 10th percentile for T10) relative to the reference period (1961 to 1990), and it is the aggregated value on gridded data at the resolution of 2.5 by 2.5 degrees latitude and longitude. We refer to [24, 25] for extensional studies on the interplay of climate changes, health risks, insurance, agriculture and macroeconomics. In this study, we used the monthly T90 and T10 with unsmoothed and unstandardized values.

**Fig. 1:**
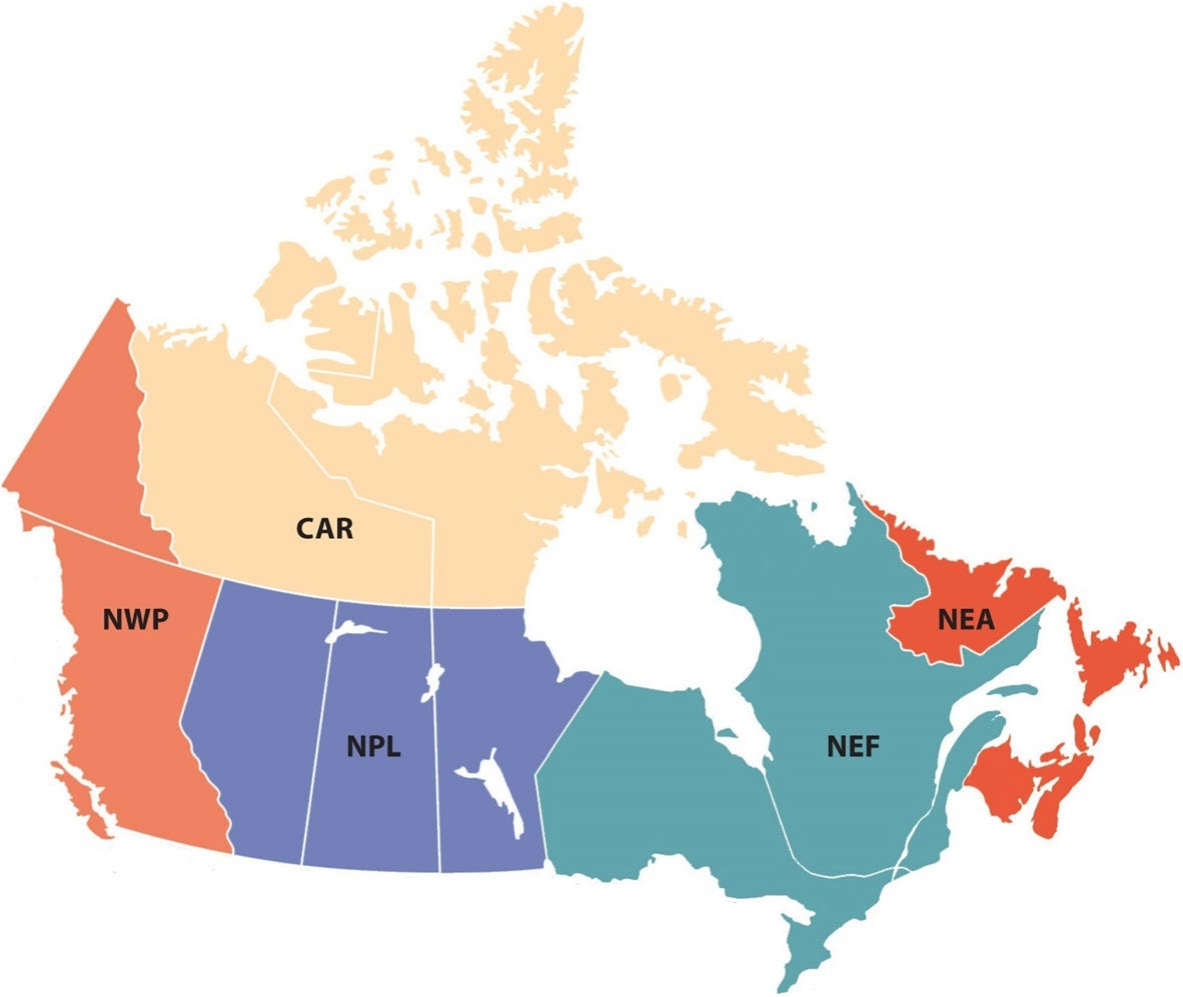
Five regions in Canada used in the ACI: Central Arctic (CAR), Northeast Atlantic (NEA), Northeast Forest (NEF), Northwest Pacific (NWP), and Northern Plains (NPL). Sources: https://actuariesclimateindex.org/data/region-definitions/

**Fig. 2:**
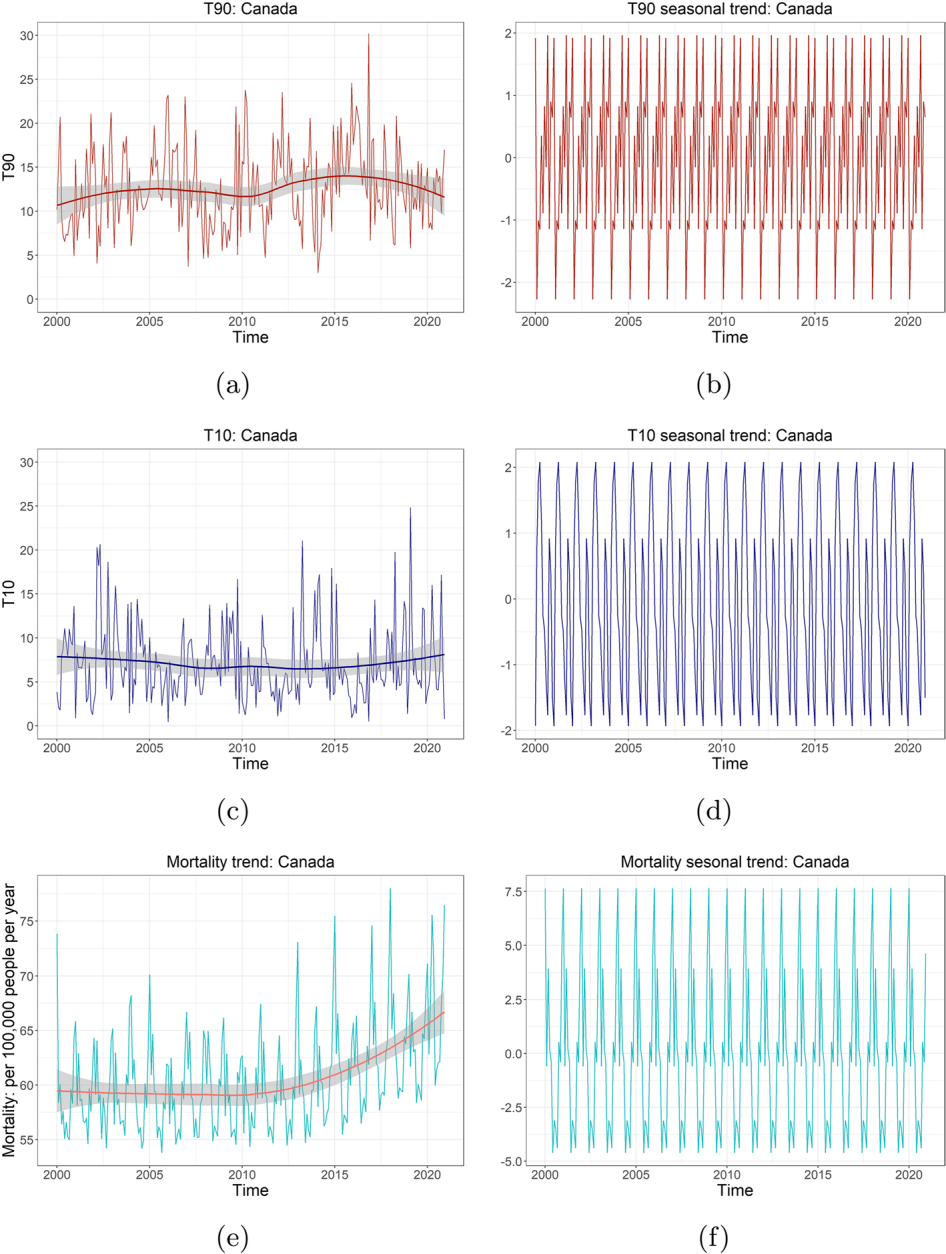
Original temporal trends (**a**, **c**, **e**) and seasonality (**b**, **d**, **f**) for T90, T10 and mortality per 100,000 in Canada during 2000–2020. The smooth lines in (**a**, **c**, **e**) shows the general trends of the data collected. The seasonality in (**b**, **d**, **f**) is from the decomposition of the original time series

**Fig. 3:**
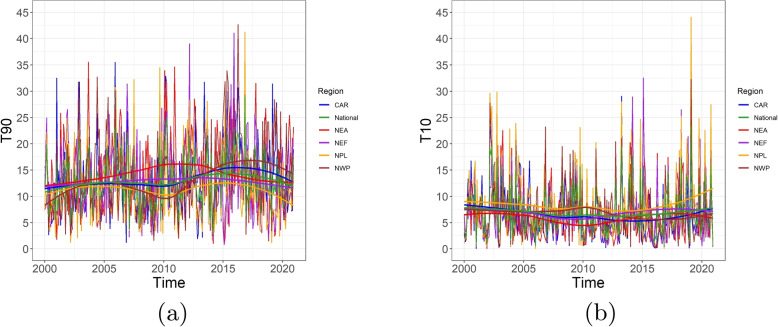
Monthly T90 (**a**) and T10 (**b**) compared among the five regions in Canada: Central Arctic (CAR), Northeast Atlantic (NEA), Northeast Forest (NEF), Northwest Pacific (NWP), and Northern Plains (NPL) in Canada. Sources: https://actuariesclimateindex.org/data/region-definitions/ for five regions

**Fig. 4:**
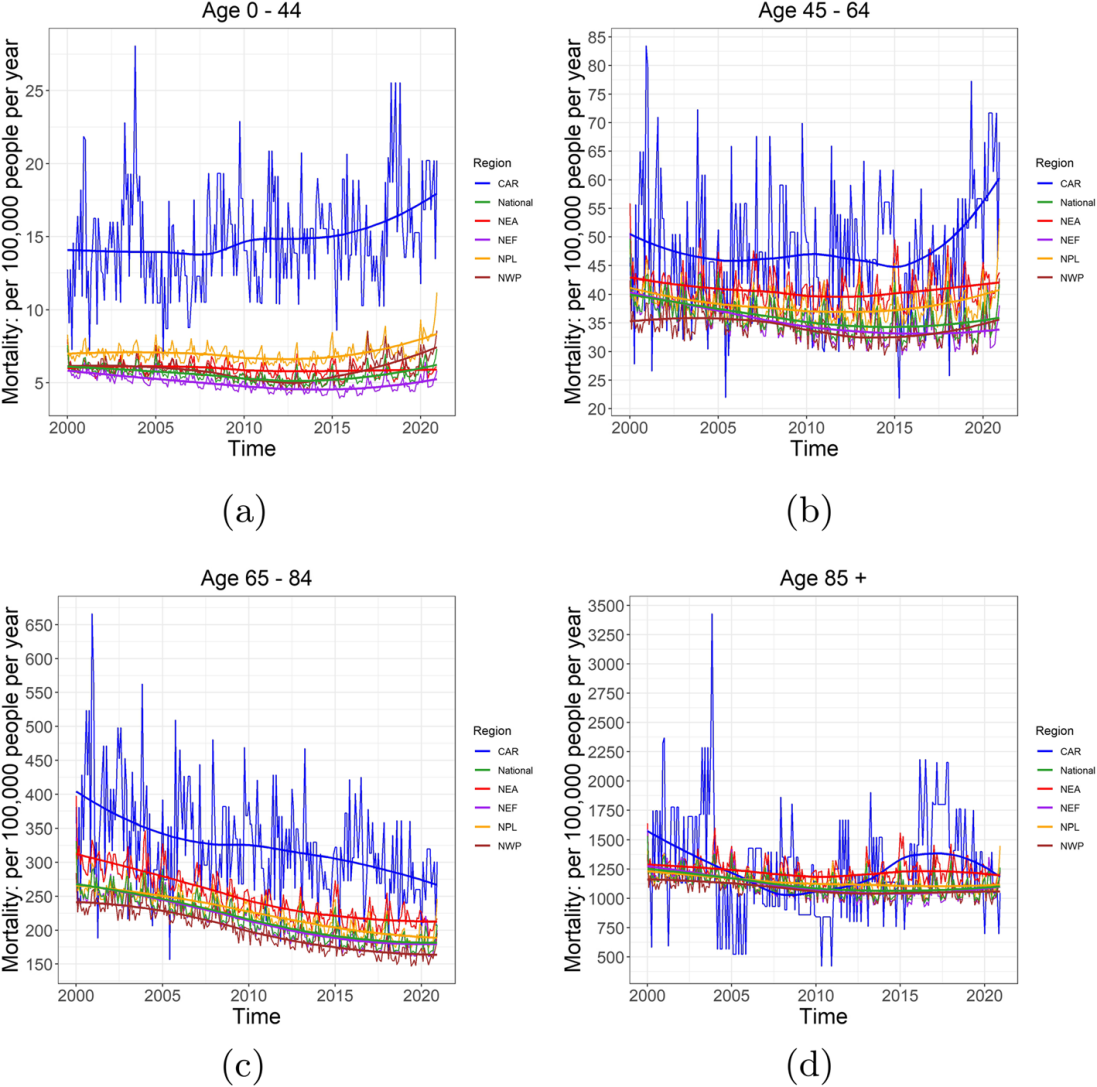
Monthly mortality per 100,000 of age groups 0–44, 45–64, 65–84 and 85+ in (**a**-**d**) compared among the five regions in Canada: Central Arctic (CAR), Northeast Atlantic (NEA), Northeast Forest (NEF), Northwest Pacific (NWP), and Northern Plains (NPL). Sources: https://actuariesclimateindex.org/data/region-definitions/ for five regions

We considered five continental regions in Canada used in the ACI, namely Central Arctic (CAR), Northeast Atlantic (NEA), Northeast Forest (NEF), Northwest Pacific (NWP), and Northern Plains (NPL); these regions are defined along state and provincial borders (Fig. 1). Since the monthly mortality data for specified age groups at the regional level are not publicly available, we assumed that the overall age structure of population and deaths did not change greatly annually, and then adopted the annual population and deaths age structure so as to obtain the monthly mortality per 100,000 people for five regions and different age groups. Provincial-level death data by month were obtained from Statistics Canada^2^, while the annual provisional death counts for four age groups of 0–44, 45–64, 65–84 and 85+ (commonly used for statistics and analysis [26]), were collected from the Canadian Vital Statistics Deaths Database^3^ (data for Yukon are not available as of 2017). The provisional annual estimates of population by age group were obtained through Statistics Canada’s Demographic Estimates program^4^.

Figure 2 shows the temporal trends (a,c,e) and seasonality (b,d,f) in the monthly T90, T10, and mortality per 100,000 people at the national level between 2000 and 2020. Figure 2a demonstrates a long-term upward trend in monthly T90 before 2016 and a downward trend afterwards, while Fig. 2c shows that T10 remains relatively stable over 20 years. The overall upward trend in T90 (except 2010), indicates that extreme high temperature becomes more frequent due to the warmer climate. This change is expected to continue in the future [27]. This is consistent with the findings of a general increase in the heat extremes frequency [28]. Figure 2e shows an upward trend of national mortality after 2012, probably attributed to population growth and population ageing, as well as the increasing exposure to unusual temperature in general (cf. Fig. 2a, c). Clear seasonality features of T90, T10 and mortality could be observed by Fig. 2b, d, f. Similar results are demonstrated for all five regions in Fig. 3.

Figure 4 shows the trends of monthly mortality per 100,000 people for four age groups at both national and regional levels in Canada. We see that the overall monthly crude mortality has increased over 20 years and the age group 85+ has higher mortality than all the other age groups, roughly four-fold of 65–84 group, and 40 fold of 45–64 group. For the age groups 0–44 and 45–64, a slightly decreasing trend in mortality is observed in NEF and NEA over 20 years, whereas the mortality has experienced an increase in CAR, NPL and NWP since 2015. For the age groups 65–84 and 85+, there are noticeable declines in all regions. Moreover, seasonal patterns are also observed with higher mortality in winter (November, December and January) and lower mortality in summer (June, July and August).

## Methodology

In this section, we introduce seasonal ARIMA models in “ARIMA model” section to adjust the seasonality and tendency of the data involved. Then, we introduce the extreme value theory, including the details of the bivariate POT method in “Extreme value theory” section.

### ARIMA model

Seasonal variation of mortality was discovered with association with socioeconomic status and outdoor temperature due to the nature of the climate change and health vulnerability [29, 30]. The seasonality usually causes the series to be non-stationary. To remove the possible seasonality and tendency of the data, the seasonal ARIMA model is applied [31]. It is one of the most commonly used statistical models to deal with non-stationary time series, with three key components: auto-regression (AR), integration (I) for non-stationary data, and current and previous residual series moving average (MA). ARIMA(*p*, *d*, *q*) with orders *p*, *d*, *q*, is defined by

$$\begin{aligned} y_{t}=c+\phi _{1} y_{t-1}+\cdots +\phi _{p} y_{t-p}+\theta _{1} \varepsilon _{t-1}+\cdots +\theta _{q}\varepsilon _{t-q}+\varepsilon _{t}\,, \end{aligned}$$

where $y_{t}$ is the differenced data, *c* is the constant term, $\phi _1, \ldots , \phi _{p}$ and $\theta _1, \ldots , \theta _{q}$ are parameters, $\varepsilon _{t}$ is the white noise with zero mean and constant variance, and *p*, *d* and *q* denote the order of the AR model, the order of differencing, and the order of the MA model, respectively. AR and MA are two widely used linear models that work on stationary time series. A seasonal ARIMA model incorporates both non-seasonal and additional seasonal factors in the ARIMA model, denoted by

1 $$\begin{aligned} \textrm{ARIMA}(p, d, q) \times (P, D, Q)_{S}\,, \end{aligned}$$

where *P*, *D* and *Q* have identical meanings that are in the non-seasonal part of the model, but involve the time span of the seasonality *S*. In our case, we set *S* to be 12 for monthly data. The residuals of seasonal ARIMA models are expected to be “white noise” (i.e., independent and identically distributed), which will be confirmed by the Box-Pierce and Ljung-Box tests at 5% significance for the optimal ARIMA model for T90, T10 and mortality in terms of the Akaike information criterion (AIC).

The general process of fitting seasonal ARIMA models is based on the Box-Jenkins method [31]. When dealing with time series data that exhibit strong seasonality and are non-stationary, we first perform differencing to make the data stationary, and then apply both Box-Pierce and Ljung-Box tests at 5% significance, to examine whether data behaves like white noise. In particular, we utilize the auto.arima() function from the **forecast** R-package [32] to find the optimal model with the lowest AIC. Then, we obtain the residuals of the optimal model by arima() function in **stats** package [33], and apply both Box-Pierce and Ljung-Box tests with Box.test() function. If the residuals fail to meet the criterion for white noise (i.e., *p*-value is less than 0.05), we proceed to fit a generalized auto-regressive conditional heteroscedasticity (GARCH) model with garch() function in **tseries** package [34]. Again, we perform the aforementioned two tests on the residuals of the GARCH model to ensure that they exhibit characteristics of white noise.

### Extreme value theory

Extreme value theory (EVT) is widely applied in the study of rare events with extreme influence on economics, finance, environment and public health [15]. Suppose that $X_1, X_2,\ldots , X_n$ is a random sample from parent $X\sim F(x)$, i.e., $X_i$’s are independent and identically distributed with common distribution function (df) *F*(*x*). Given a large threshold *u*, the distribution $F_u(y)$ of the excess $Y_{[u]}=X -u\mid X>u$, is thus given by

$$\begin{aligned} F_u(y)=\mathbb {P} \left\{ X-u \le y \mid X>u\right\} =\frac{\mathbb {P} \left\{ u<X \le y+u\right\} }{\mathbb {P} \left\{ X>u\right\} }=\frac{F(y+u)-F(u)}{1-F(u)}. \end{aligned}$$

The $F_u(y)$ can be approximated by generalized Pareto (GP) distribution $G_{\xi , \sigma }(y) = 1 - (1+\xi y/\sigma )_+^{-1/\xi },y>0$ for sufficiently high threshold [15]. Therefore, the tail distribution function $\overline{F}(x) = 1-F(x)$ of *X* can be approximated by

2 $$\begin{aligned} \overline{F}(x)=\zeta _u \overline{F}_u(x-u) \approx \zeta _u \overline{G}_{\xi ,\sigma }(x-u), \quad {x}>u. \end{aligned}$$

Here $\zeta _u = \overline{F}(u)$, and $\xi \in \mathbb {R}$ and $\sigma >0$ are the shape/tail and scale parameters of GP distribution, respectively. In practice, the exceedance probability $\overline{F}(x)$ gives insights into the potential risk, and a larger tail risk is indicated by a larger tail index $\xi$. Its estimate can be obtained through the extrapolation approach via Eq.(2): to get the approximated tail probability of the GP model using the maximum likelihood estimation of $\xi ,\sigma$ based on excesses $(x_{(i)}-u)'s$ with $x_{(1)}\ge \cdots \ge x_{(n_u)}$ exceeding the threshold *u* and the estimate of $\zeta _u$ as $n_u/n$. Theoretically, the threshold *u* can be determined by minimizing the mean square error of the Hill estimator of $\xi$, balancing the model bias and variance. A common graphical approach in the determination of the threshold is to check both the linearity of the empirical mean excess function and the stability estimation plots of both scale and shape parameters, as illustrated by Appendix Figure A.3.

**Bivariate POT method**. Many problems involving extreme events are inherently multivariate. A fundamental issue thus arising is how extremes in one variable relate to those in another. Dependence occurs if the process is studied at neighbouring spatial locations during its temporal evolution or shares common meteorological conditions. The study of multivariate extremes splits apart into two steps: the marginal analysis first and then the dependence measures, which are commonly supposed to be marginally invariant. Commonly, one may employ marginal tail information to transform the marginal distribution to a common scale, e.g., unit Fréchet distribution. Let $(X_i,Y_i)$ be a random sample of size *n* with common distribution function (d.f.) *F*(*x*, *y*). Assume its marginal $F_X$ and $F_Y$ satisfy Eq.(2) on regions of the form $x> u_x$ and $y> u_y$ for large enough thresholds $u_x$ and $u_y$, with respective parameter sets $(\zeta _x, \sigma _x, \xi _x)$ and $(\zeta _y, \sigma _y, \xi _y)$. Denote by

3 $$\begin{aligned} \left\{ \begin{array}{l} \widetilde{X}_i = -\left( \log \left\{ 1-\zeta _x\left[ 1+\frac{\xi _x\left( X_i - u_x\right) }{\sigma _x}\right] ^{-1 / \xi _x}\right\} \right) ^{-1}, \\ \widetilde{Y}_i = -\left( \log \left\{ 1-\zeta _y\left[ 1+\frac{\xi _y\left( Y_i - u_y\right) }{\sigma _y}\right] ^{-1 / \xi _y}\right\} \right) ^{-1}. \end{array} \right. \end{aligned}$$

Thus, $(\widetilde{X}_i, \widetilde{Y}_i)$ follows joint d.f. $\widetilde{F}$ with unit Fréchet margins $\exp \left( -\widetilde{x}^{-1}\right) , \widetilde{x}>0$ and

$$\begin{aligned} \widetilde{F}(\widetilde{x}, \widetilde{y}) = F(x, y),\quad x>u_x,\, y> u_y. \end{aligned}$$

Suppose that *F* is in the max-domain of attraction of a bivariate extreme value distribution, i.e., the normalized component-wise maxima of observation from *F* follow asymptotically a bivariate extreme value distribution. This is equivalent to

$$\begin{aligned} F(x, y) = \left\{ \widetilde{F}^n(\widetilde{x}, \widetilde{y})\right\} ^{1/n} \approx G(\widetilde{x}, \widetilde{y}), \quad x> u_x,\, y> u_y, \end{aligned}$$

where $\widetilde{x}$ and $\widetilde{y}$ are defined in terms of *x* and *y* by Eq.(3) and *G* is a bivariate d.f. with unit Fréchet margins ([15], Chapter 8). Note that *G* has no parametric form. In our context, we take a flexible logistic model for our bivariate statistical analysis, given by

$$\begin{aligned} G(\widetilde{x}, \widetilde{y}) = \exp \left( -(\widetilde{x}^{-1/\alpha } + \widetilde{y}^{-1/\alpha })^{\alpha }\right) \end{aligned}$$

with dependence parameter $\alpha \in (0, 1]$. The limiting case of $\alpha \rightarrow 0$ corresponds to the variables being total dependent. That is

$$\begin{aligned} G(\widetilde{x}, \widetilde{y}) \rightarrow \exp \left( -\textrm{max}(\widetilde{x}^{-1}, \widetilde{y}^{-1})\right) ,\quad \text{ as } \alpha \rightarrow 0. \end{aligned}$$

As $\alpha$ increases the dependence becomes weak, and when $\alpha = 1$, the variables are independent: $G(\widetilde{x}, \widetilde{y}) = \exp \left( -1/\widetilde{x} -1/ \widetilde{y}\right)$. Hence, the bivariate logistic model covers all levels of dependence, from independence to perfect dependence.

One of the most useful alternative approaches to describe the dependence structure of *G* is extreme tail index $\chi \in [0,1]$, giving a rough but representative picture of the full dependence structure. The extreme tail index is a limiting measure of the tendency for one variable to be large conditional on the other variable being large, i.e.,

$$\begin{aligned} \chi = \underset{u\rightarrow 1}{\textrm{lim}} \mathbb {P} \left\{ F_Y(Y)> u \mid F_X(X) > u\right\} , \end{aligned}$$

where $F_X$ and $F_Y$ are the marginal d.f.s of *X* and *Y*. We see that, the index $\chi$ describes the likelihood that the quantity *X* (here the excess high or low temperature frequency) becomes so large as to cause *Y* (here the excess mortality) to experience such a tail event at least as severe (in quantile terms) as *X*, ranging in [0, 1]. In particular, the variables are said to be asymptotically independent when $\chi =0$, while $\chi =1$ corresponds to the total dependence.

Another alternative approach for extremal dependence is to develop functions that give a complete characterization of the extremal dependence, like the spectral distribution function ([35]), the Pickands dependence function [36] or the stable tail dependence function [37]. These functions can be seen as the analogues of copulas in classical statistics. In this paper, we will utilize the *Pickands dependence function*

$$\begin{aligned} A(\omega ) = -\textrm{log}\, G(1/(1-\omega ), 1/\omega ): [0,1] \mapsto [0,1], \end{aligned}$$

which equals $(\omega ^{1/\alpha } + (1-\omega )^{1/\alpha })^{\alpha }$ for the bivariate logistic model. We have

$$\begin{aligned} \textrm{max}(\omega , 1-\omega ) \le A(\omega ) \le 1,\quad \omega \in (0,1). \end{aligned}$$

Note that the lower and upper bounds of the Pickands dependence function above correspond to the total dependence and independence, respectively. Therefore, we can show graphically the dependence by examining the closeness of the lower bound of the Pickands dependence function.

With white-noise residuals obtained from the ARIMA/ARIMA-GARCH models, we employ the bivariate POT method with implementation in the **POT** R-package [38]. The marginal thresholds ($u_x, u_y$) can be selected by empirical mean residual life plots and scale/shape parameters’ stability plots. These plots are generated using the mrlplot() and tcplot() functions, respectively. Then we proceed to fit the joint residuals by bivariate GP distribution with the logistic dependence structure, implemented by fitbvgpd() function. The extreme tail index $\chi$ is subsequently calculated based on the reported $\alpha$ in the bivariate logistic model, and the Pickands dependence function is visualized by the pickdep() function.

## Results

The bivariate POT method requires data to be independent and identically distributed, thus the ARIMA model is applied to adjust the seasonality, trends and non-stationarity from temperature frequency and mortality time series. We first fit monthly T90, T10 and mortality data by seasonal ARIMA models and perform the Box-Pierce and Ljung-Box tests on the residuals of the optimal ARIMA model in terms of AIC values (“Results of ARIMA models” section). We then present the main results of bivariate POT analysis for residuals of T90, T10 and mortality per 100,000 people (“Results of bivariate POT models” section).

### Results of ARIMA models

Table 1 shows the parameters involved for the optimal ARIMA model based on AIC values. Since the raw data is based on month, we set the order of seasonal difference $S=12$ and $D=1$ in the seasonal part of the ARIMA model.

The obtained residuals from the ARIMA model were considered in this context as *excess T90, T10 or mortality* since its trends and seasonality were adjusted and its white noise features were confirmed according to the stationary test at 5% significance, except those for age group 45-64 in NWP, for 65-84 in NEA, NEF and NWP. In these cases, GARCH(1,0) was further conducted [39].

Note that a larger dispersion of residuals for elderly groups (aged 65+) was observed (Appendix Figure A.1 and Table A.1, the mean and standard deviation of relevant residuals). This might be reasonable since elderly people may face a higher mortality risk due to confounding variables beyond extreme temperatures. In addition, larger uncertainty of the mortality was observed in CAR from the larger standard deviation. We thus presented the scaled residuals plots which showed similar dispersion in all age groups and regions, but some large dispersion in May and June 2020 in all regions and age groups (Appendix Figure A.2), probably caused by the outbreak of the COVID-19.

### Results of bivariate POT models

To conduct the bivariate extreme analysis, we need to select a proper threshold with a trade-off between bias and variance. One illustrating example is given for threshold selection in Appendix Figure A.3, namely, for national T90, the 70% quantile $u=3.67$ is chosen since the mean excess values are approximately linear around it, and parameters’ estimations are also stable with subsequent values having higher variance due to smaller number of exceedances. For simplicity, we set the threshold to be the 70% quantiles for residuals of both temperature and mortality data, and the resulting threshold-excesses follow GP distribution using KS test at 5% significance.

The marginal distributions are thus transformed to be a common unit Fréchet margins based on calculated scale and tail parameter sets given in Appendix Table A.2. We observe relatively heavy tails (with tail indices exceeding 0.10) for mortality rates in NEF and NPL, suggesting a higher likelihood of extremely severe mortality events in these regions. Meanwhile, the heavy tail indices for T10 in NEF and T90 in NPL indicate increasing potentials for experiencing exceptionally frequent cold or hot days.

**Table 1:** Seasonal ARIMA models for T90, T10 and mortality per 100,000 people per age group at both national and regional levels: Central Arctic (CAR), Northeast Atlantic (NEA), Northeast Forest (NEF), Northwest Pacific (NWP), and Northern Plains (NPL) in Canada. Sources: https://actuariesclimateindex.org/data/region-definitions/ for five regions based on AIC

Region	T90		T10		0–44	
	(*p*, *d*, *q*)	(*P*, *D*, *Q*)	(*p*, *d*, *q*)	(*P*, *D*, *Q*)	(*p*, *d*, *q*)	(*P*, *D*, *Q*)
National	(1, 0, 1)	(2, 1, 2)	(1, 0, 1)	(2, 1, 2)	(2, 1, 1)	(0, 1, 2)
CAR	(1, 0, 0)	(1, 1, 0)	(0, 0, 1)	(2, 1, 2)	(2, 0, 2)	(1, 1, 0)
NEA	(1, 0, 0)	(1, 1, 0)	(1, 0, 1)	(2, 1, 0)	(2, 0, 0)	(1, 1, 0)
NEF	(1, 0, 0)	(1, 1, 0)	(1, 0, 1)	(2, 1, 2)	(2, 1, 1)	(1, 1, 2)
NPL	(0, 0, 1)	(2, 1, 0)	(0, 0, 1)	(1, 1, 0)	(1, 1, 1)	(1, 1, 1)
NWP	(1, 0, 1)	(1, 1, 0)	(0, 0, 1)	(1, 1, 0)	(1, 1, 1)	(0, 1, 1)
Region	45–64		65–84		85+	
	(*p*, *d*, *q*)	(*P*, *D*, *Q*)	(*p*, *d*, *q*)	(*P*, *D*, *Q*)	(*p*, *d*, *q*)	(*P*, *D*, *Q*)
National	(3, 1, 1)	(0, 1, 2)	(2, 0, 3)	(1, 1, 2)	(3, 1, 2)	(0, 1, 2)
CAR	(2, 0, 1)	(2, 1, 2)	(4, 0, 3)	(1, 1, 0)	(1, 0, 1)	(2, 1, 2)
NEA	(1, 0, 0)	(2, 1, 0)	(1, 0, 1)	(2, 1, 1)	(2, 0, 1)	(1, 1, 0)
NEF	(2, 1, 2)	(1, 1, 2)	(2, 0, 0)	(1, 1, 2)	(2, 1, 1)	(1, 1, 2)
NPL	(1, 1, 1)	(1, 1, 2)	(1, 0, 0)	(1, 1, 0)	(1, 0, 0)	(1, 1, 0)
NWP	(1, 0, 0)	(0, 1, 1)	(1, 0, 0)	(1, 1, 0)	(0, 0, 1)	(0, 1, 1)

Table 2 lists the obtained tail dependence index $\chi$ together with the marginal thresholds used, the joint exceeding proportion for T90/T10 and mortality rate. The statistical significance from independence ($\chi >0$), equivalent to $\alpha <1$, is assessed by the estimate of $\alpha$ and its 95% confidence interval. In general, the extremal dependence between the frequency of low temperature (T10) and mortality is relatively strong with almost all $\chi$ greater than 0.10. In other words, it is likely (more than 10%) to occur with excess mortality if people are exposed to an unusual cold temperature with extreme intensity. In other words, once the Canadians experience even more frequent cold snaps, the concurrent mortality is more likely to increase. This extremal dependence was also observed from the empirical estimation of $\chi$ at quantile levels 0.75, 0.80 and 0.85 for T10 with all greater than 0.15 (Appendix Table A.3).

**Table 2:** Results from bivariate POT analysis on T90/T10 and mortality per 100,000 people at both national and regional levels. Values in bold indicates statistically significant ($\chi >0$) at 5% level. Five regions: Central Arctic (CAR), Northeast Atlantic (NEA), Northeast Forest (NEF), Northwest Pacific (NWP), and Northern Plains (NPL) in Canada. Sources: https://actuariesclimateindex.org/data/region-definitions/

Region	0–44			45–64			65–84			85+		
	Threshold		$\chi$	Threshold		$\chi$	Threshold		$\chi$	Threshold		$\chi$
	Marginal	Joint (%)		Marginal	Joint (%)		Marginal	Joint (%)		Marginal	Joint (%)	
T90 and Mortality per 100,000												
National	(3.67, 5.82)	0.0794	0.0013	(3.67, 0.365)	0.0714	0.0011	(3.67, 1, 93)	0.0873	0.0013	(3.67, 10.9)	0.0635	<0.0000
CAR	(3.82, 1.89)	0.0833	0.0012	(3.82, 4.52)	0.0794	0.0009	(3.82, 30)	0.1071	0.0417	(3.82, 137)	0.0833	0.0082
NEA	(3.16, 0.094)	0.0714	0.0007	(3.16, 0.732)	0.0675	0.0011	(3.16, 0.465)	0.0797	0.0010	(3.16, 25.1)	0.0794	0.0011
NEF	(3.52, 0.0675)	0.0873	0.0006	(3.52, 0.439)	0.0873	0.0013	(3.52, 0.139)	0.0677	0.0003	(3.52, 14)	0.0992	0.0000
NPL	(2.59, 0.133)	0.0913	0.0002	(2.59, 0.66)	0.0714	0.0008	(2.59, 1.24)	0.0635	0.0008	(2.59, 13.1)	0.0635	0.0013
NWP	(3.43, 0.119)	0.0992	**0.3182**	(3.43, 0.247)	0.0916	**0.3182**	(3.43, 0.079)	0.0916	**0.3182**	(3.43, 7.9)	0.1032	**0.3182**
T10 and Mortality per 100,000												
National	(0.79, 5.82)	0.0913	0.0497	(0.79, 0.37)	0.119	0.096	(0.79, 1.93)	0.119	0.1124	(0.79, 10.90)	0.127	0.1209
CAR	(1.06, 1.89)	0.1111	**0.3182**	(1.06, 4.52)	0.1151	**0.3182**	(1.06, 30)	0.0873	**0.3182**	(1.06, 137)	0.0992	**0.3182**
NEA	(1.32, 0.094)	0.1270	0.0726	(1.32, 0.732)	0.1310	**0.1323**	(1.32, 0.465)	0.1076	0.0707	(1.32, 25.1)	0.1389	**0.1768**
NEF	(1.17, 0.068)	0.1270	**0.1341**	(1.17, 0.439)	0.1230	**0.1469**	(1.17, 0.139)	0.1315	**0.1455**	(1.17, 14)	0.1190	**0.1346**
NPL	(2.44, 0.133)	0.1111	0.0623	(2.44, 0.66)	0.1190	0.1096	(2.44, 1.24)	0.1349	**0.1886**	(2.44, 13.1)	0.1270	**0.1625**
NWP	(1.46, 0.119)	0.1032	0.0484	(1.46, 0.247)	0.1116	**0.3182**	(1.46, 0.079)	0.1036	0.0835	(1.46, 7.9)	0.1071	0.1122

**Fig. 5:**
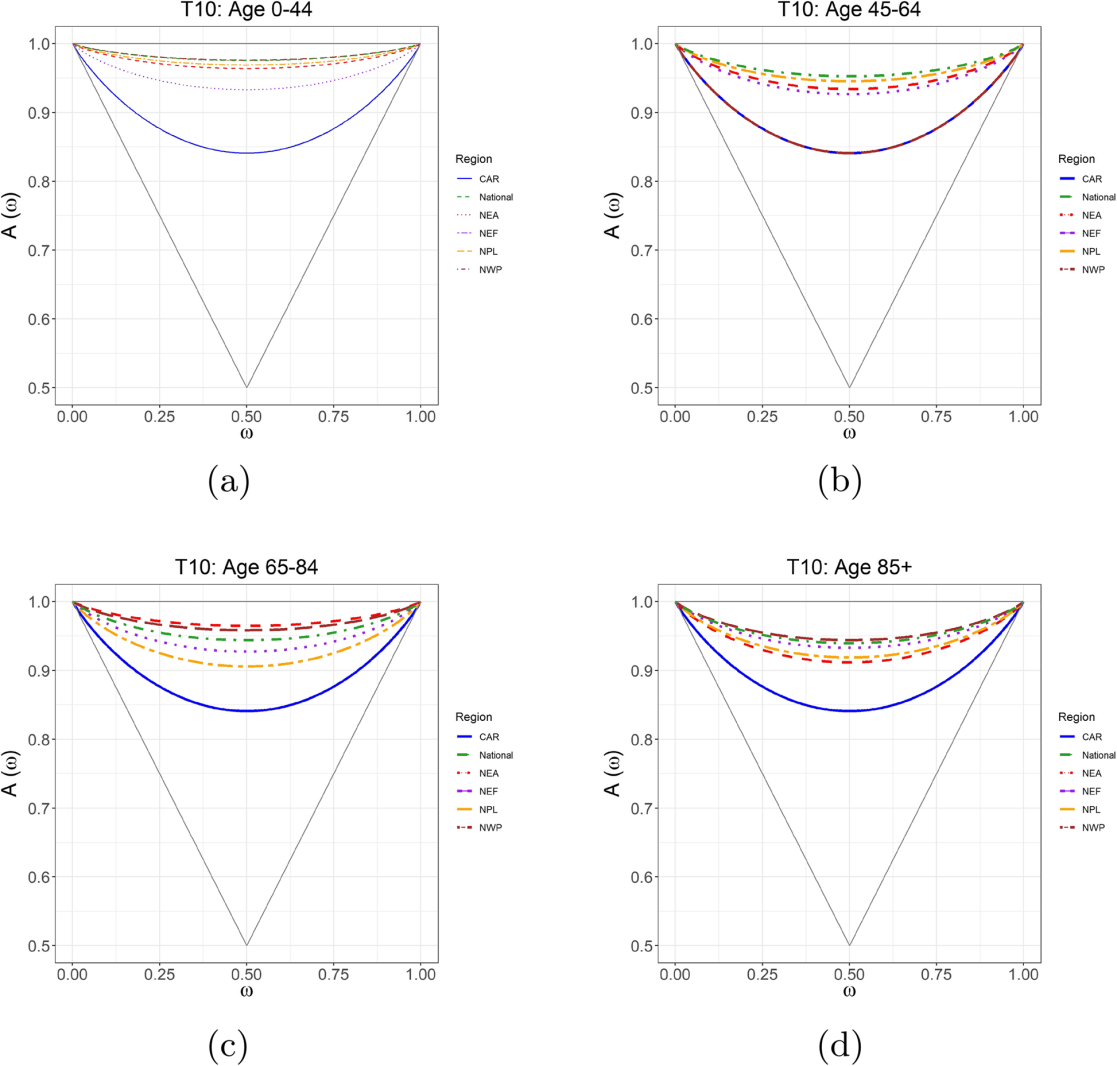
Pickands dependence functions for T10 and mortality per 100,000 of age groups 0–44, 45–64, 65–84 and 85+ in (**a**-**d**) compared among Canada and the five contingent regions: Central Arctic (CAR), Northeast Atlantic (NEA), Northeast Forest (NEF), Northwest Pacific (NWP), and Northern Plains (NPL). Sources: https://actuariesclimateindex.org/data/region-definitions/ for five regions

**Fig. 6:**
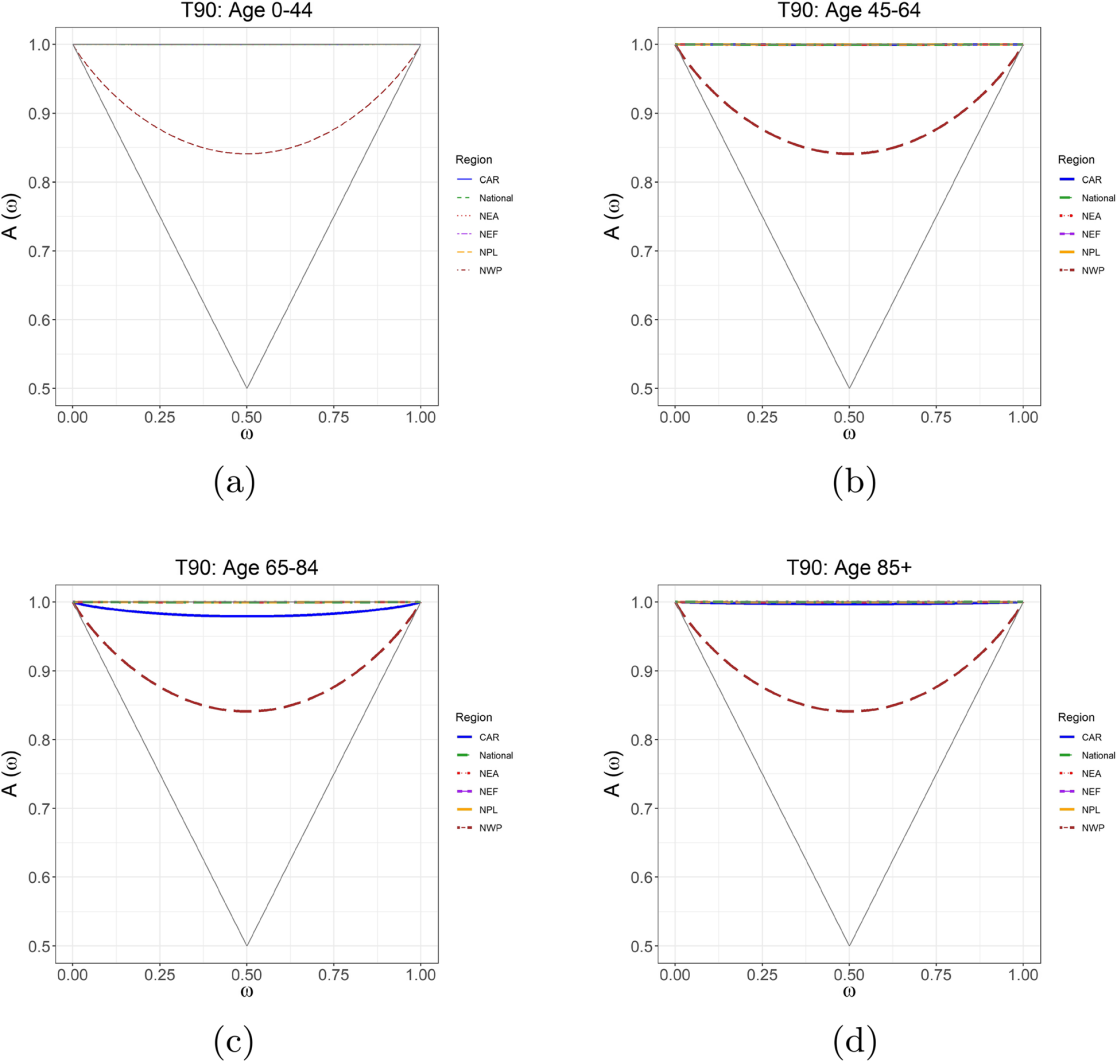
Pickands dependence functions for T90 and mortality per 100,000 of age groups 0–44, 45–64, 65–84 and 85+ in (**a**-**d**) compared among Canada and the five contingent regions: Central Arctic (CAR), Northeast Atlantic (NEA), Northeast Forest (NEF), Northwest Pacific (NWP), and Northern Plains (NPL). Sources: https://actuariesclimateindex.org/data/region-definitions/ for five regions

The extremal dependence between high/low temperature and mortality varies across age groups and across regions, and the dependence is considered as fairly weak if $\chi$ is close to zero. Our results show weak dependence between extreme high temperature (T90) and mortality in age groups 0–44 and 65–84 in NEA, NEF and NPL, age group 45–64 in CAR and NPL, and age group 85+ in NEF, since $\chi$ is less than or equals 0.001. It indicates that the health of those people is less likely to be affected even if they experience an extremely high-temperature frequency. Among all five regions, NWP has the highest overall extremal dependence ($\chi =0.3182$) and NEF has the lowest overall extremal dependence.

Pickands dependence function plots, as a visualizing tool of dependence strength, are given in Figs. 5 and 6 for T10 and T90, respectively. In each triangle, the horizontal line at the top indicates the independence, and the other two lines forming the triangle indicate the full dependence. The colored curves represent estimated Pickands extremal dependence. The differences in the strength of the extremal dependence between T90 and mortality for most regions (expect for NPL) are minor. NPL (in the dark red dashed line) is far from the horizontal line of the triangle, while other lines almost overlap with the horizontal line, indicating the same finding that NPL has the strongest extremal dependence for all age groups, which is consistent with the results in Table 2.

Figure 5 shows that the extremal dependence of T10 and mortality varies in age groups and regions. We see that CAR (in blue line) exhibits the strongest and significant extremal dependence for all ages among all regions. For age groups 0–44 and 85+, NWP (in dark red dashed line) demonstrates the weakest and non-significant extremal dependence, and while for age group 65–84, NEA (in red dashed line) has the weakest extremal dependence. In the age group 45–64, NWP shows the same strongest and significant extremal dependence as CAR, while NPL has relatively weaker and non-significant extremal dependence. These findings align with the significance of dependence highlighted in Table 2

To summarize, our results provide the evidence that the frequent low temperature gives a larger effect on mortality risk in Canada (except NEA, NPL and NWP for age group 0–44), while the frequent high temperature does not seem to have significant impact in most regions (except NWP). This is consistent with existing research that winter is at higher risk for temperature-related mortality, mainly because low temperatures can cause a physiological impact on the human body and increase the risk of cardiovascular, cerebrovascular, and respiratory diseases [30].

Extreme cold and heat events can both occur in Canada, while extreme cold events are generally more frequent and widespread across the country. This is due to Canada’s high latitude and the influence of polar air masses that result in long and cold winters. In contrast, concurrent extreme heat events in multi-provinces are relatively rare. However, in recent years, there has been a notable increase in the frequency and severity of extreme heat events in some parts of Canada, such as the 2021 heat dome in British Columbia and the 2010 heatwave in Quebec, due to a changing climate [40]. Our results show that the mortality in NWP (CAR) is highly associated with extreme frequency of very high (low) temperature for all age groups. This suggests that NWP and CAR are particularly vulnerable to extreme temperature events due to their unique geographic and climatic characteristics. NWP is susceptible to air dryness, low humidity, and limited rain during summer due to its location along the Pacific Ocean and several mountain ranges, while CAR is characterized by its high latitudes, harsh polar climate, and limited access to resources and services. We see also that the elderly (aged 65+) are very vulnerable to high frequencies of extreme low temperatures, and this result is consistent with existing research indicating that the elderly are at a higher risk for temperature-related mortality [41]. Even middle-aged people (aged 45–64) are also observed to be highly associated with effects of the high frequency of low temperature.

## Discussion

In this study, we examined the extreme association between the frequency of high/low temperature and mortality for different age groups in Canada. We proposed a combined bivariate peaks-over-threshold and ARIMA approach to model the joint extremes in monthly high/low temperature frequency and excess mortality, and quantified the extremal dependence between two extreme risks. Our findings reveal that extreme cold events are more frequently associated with serious mortality than extreme heat events in Canada. Neglect of the extremal dependence risks might underestimate the potential for very serious mortality events occurring in conjunction with a high frequency of cold days. This underestimation could have significant ramifications, including potentially substantial economic losses, strained healthcare systems, and most importantly, the tragic loss of human lives that might have been prevented with a more accurate modelling approach. The identified multi-provincial disparity and uneven vulnerability of age groups provide new insights for comprehensive risk management in the healthcare sector, public authorities and environmental agencies.

Quantitative analysis of the strength of extremal dependence was successfully conducted by bivariate logistic models with also Pickands dependence function plots. These plots provide a useful visual tool for modelling and displaying the dependence structures between variables based on their extremes. The dynamics of extreme temperature events and their impact on health outcomes frequently vary over time and across geographical regions. The interpretation of these extreme dependencies might be attributed to these covariates. Various tools have been developed to address this, including the conditional Pickands dependence function [36, 37], regression models for spectral density function which is an alternative and equivalent Pickands function [35, 42]. Notably, this quantitative analysis method can be promisingly applied to the study of the extreme association between natural events (e.g., air pollution, wildfire, rainfall and droughts) and public health risks (e.g., infectious diseases) as well as insurance risk management [14, 20].

The studied association between extreme temperature and excess mortality may vary in different spatial scales of investigation. The methodology can still assist in examining the temperature-mortality association in city-level and regional-specific studies, where detailed analysis can provide valuable findings and targeted recommendations for specific areas, see also e.g., [10] for relevant methods designed for data analysis at the small-area level. Other useful applications refer to the study of the impacts on cause-specific mortality, including the leading top mortality of diseases [43].

By focusing on more specific health outcomes, we can gain a deeper understanding of how extreme temperatures affect human health and develop targeted interventions to mitigate their impacts. Further interest is to examine the pixel resolution spatial analysis of the post influences of extreme temperatures on human health across different regions. This technique can be employed to assess the spatial and temporal pattern of extreme temperature events and their health impacts. Additionally, other factors such as demographic, socioeconomic, and infrastructural factors can also be considered in the future research [44]. The influences of multiple extreme meteorological variables, e.g., humidity and temperature, on the resulting diseases’ risks can be conducted at both national and county levels for a variety of diseases [45].

## Conclusion

The novelty of this paper is the development of multivariate extreme value modelling methods to identify the post-influences of various extreme weather events on public health issues. This study employs the bivariate logistic model to examine joint extremes of monthly low/high temperature frequency and excess mortality in Canada during 2000-2020. To the best of our knowledge, this is the first study to provide assessments and important insights regarding the joint distribution of temperature frequency and mortality extremes for different age groups at a regional level in Canada. Our results can help researchers, communities, and policymakers to reduce the adverse effects of more frequent and intense extreme weather events on vulnerable populations. Moreover, the multivariate extreme value theory can be a useful tool for studying the extremal dependence structure between multiple extreme natural events and consequential public health risks.

### Supplementary Information

Supplementary Material 1.

## Data Availability

No datasets were generated or analysed during the current study.

## Abbreviations

ACI: Actuaries Climate Index
AIC: Akaike information criterion
ARIMA: Auto-regressive Integrated Moving Average
CAR: Central Arctic
ETE: Extreme temperature events
EVT: Extreme value theory
GARCH: Generalized auto-regressive conditional heteroscedasticity
GP: Generalized Pareto
NEA: Northeast Atlantic
NEF: Northeast Forest
NWP: Northwest Pacific
NPL: Northern Plains
POT: Peaks-Over-Threshold
STD: Standard deviation
T10: The change in the frequency of temperature above the 10th percentile
T90: The change in the frequency of temperature above the 90th percentile

## Footnotes

1 https://actuariesclimateindex.org/ (accessed April 27, 2023)

2 https://www.statcan.gc.ca/en/start (accessed April 27, 2023)

3 https://www23.statcan.gc.ca/imdb/p2SV.pl?Function=getSurvey &SDDS=3233#a1 (accessed April 27, 2023)

4 https://www23.statcan.gc.ca/imdb/p2SV.pl?Function=getSurvey &SDDS=3604 (accessed April 27, 2023)
