# Supplementary figures and images for: Bivariate extreme value analysis of extreme temperature and mortality in Canada, 2000-2020
Source: BMC Public Health. 2024 May 18;24:1344. doi: 10.1186/s12889-024-18785-3 (PMC11102153; doi:10.1186/s12889-024-18785-3)

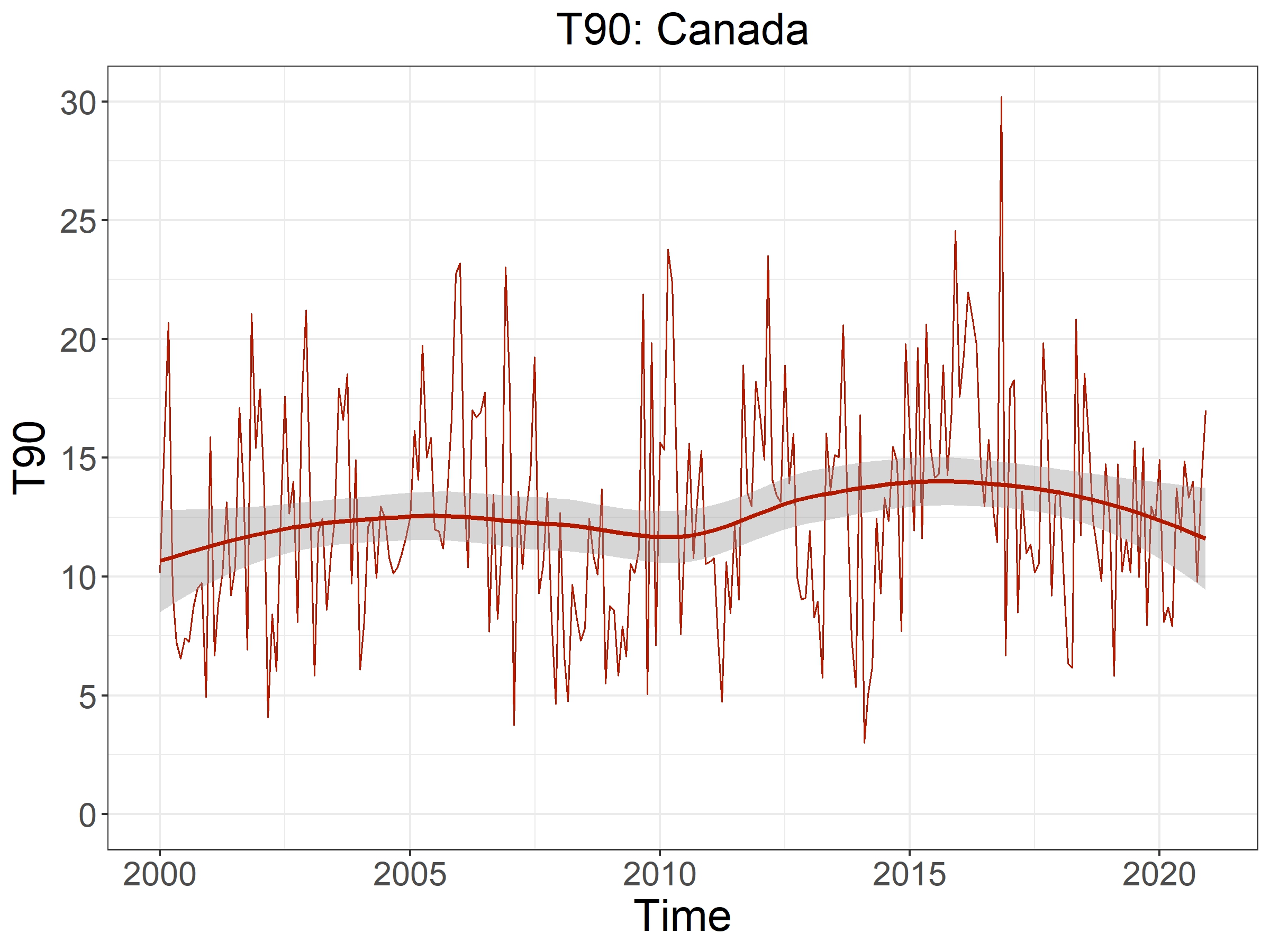

Supplement: Supplementary file 1 — Supplementary Material 1. [file 12889_2024_18785_MOESM1_ESM.zip › updated fig/Fig1_Canada_T90.jpeg]

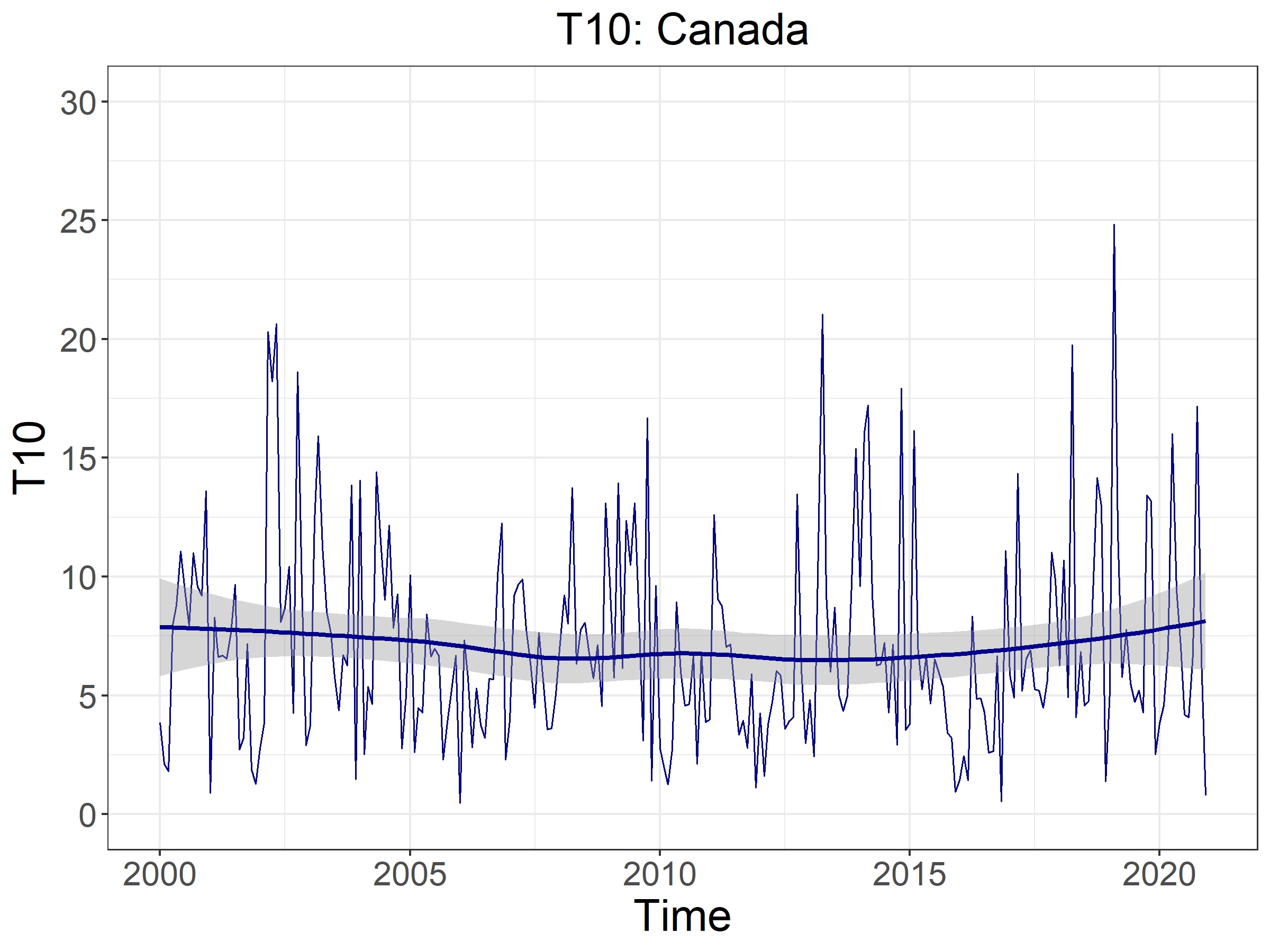

Supplement: Supplementary file 1 — Supplementary Material 1. [file 12889_2024_18785_MOESM1_ESM.zip › updated fig/Fig1_Canada_T10.jpeg]

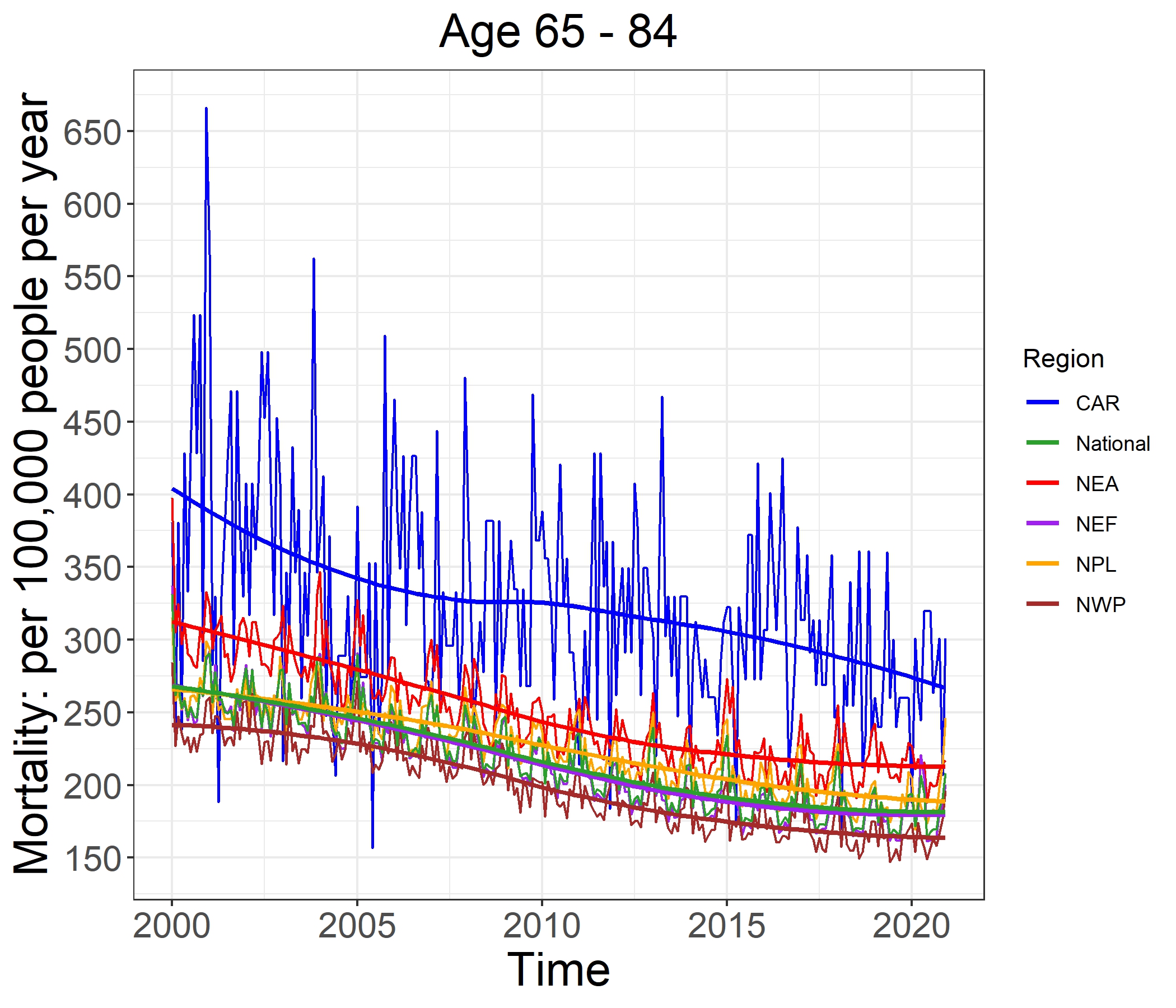

Supplement: Supplementary file 1 — Supplementary Material 1. [file 12889_2024_18785_MOESM1_ESM.zip › updated fig/mortality_6584.jpeg]

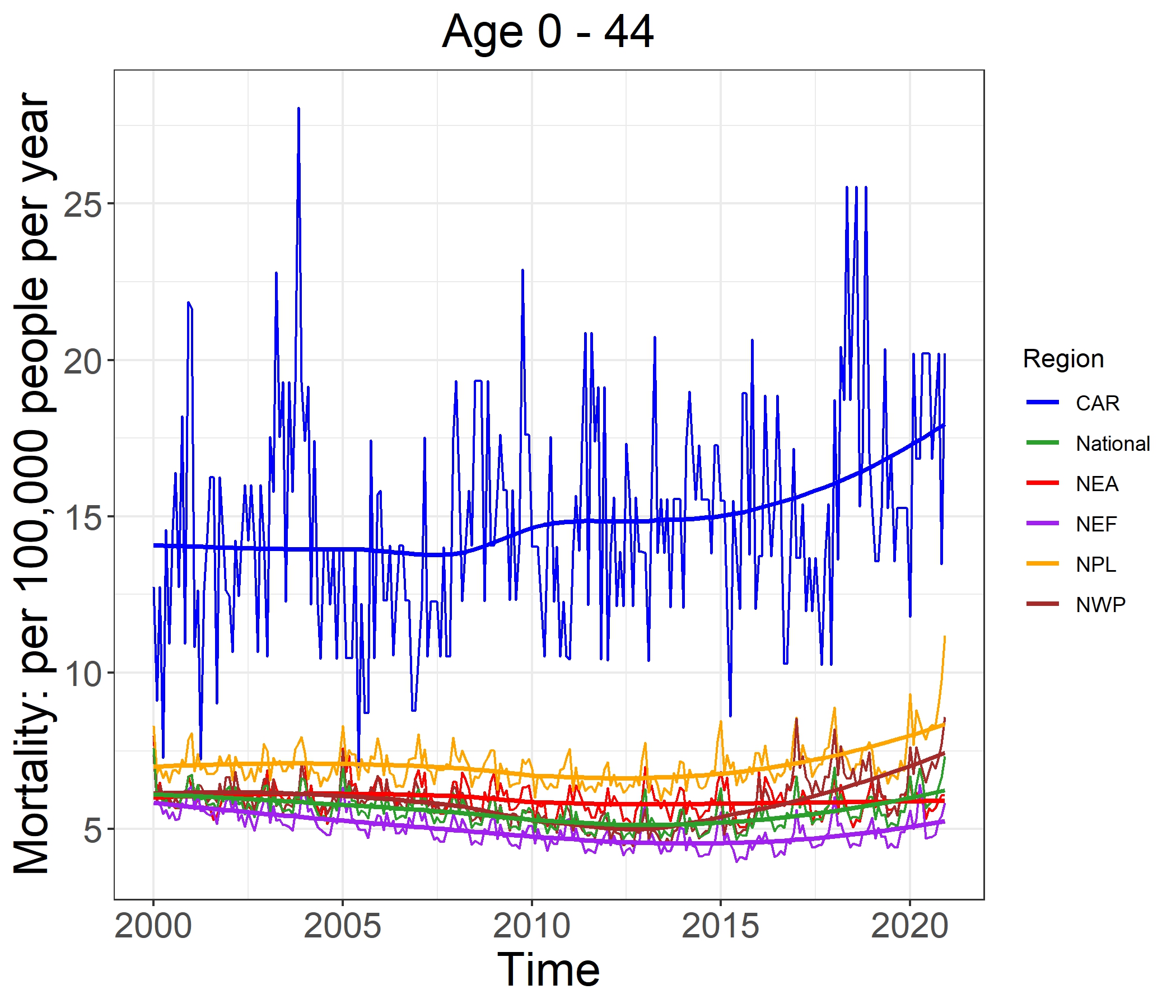

Supplement: Supplementary file 1 — Supplementary Material 1. [file 12889_2024_18785_MOESM1_ESM.zip › updated fig/mortality_044.jpeg]

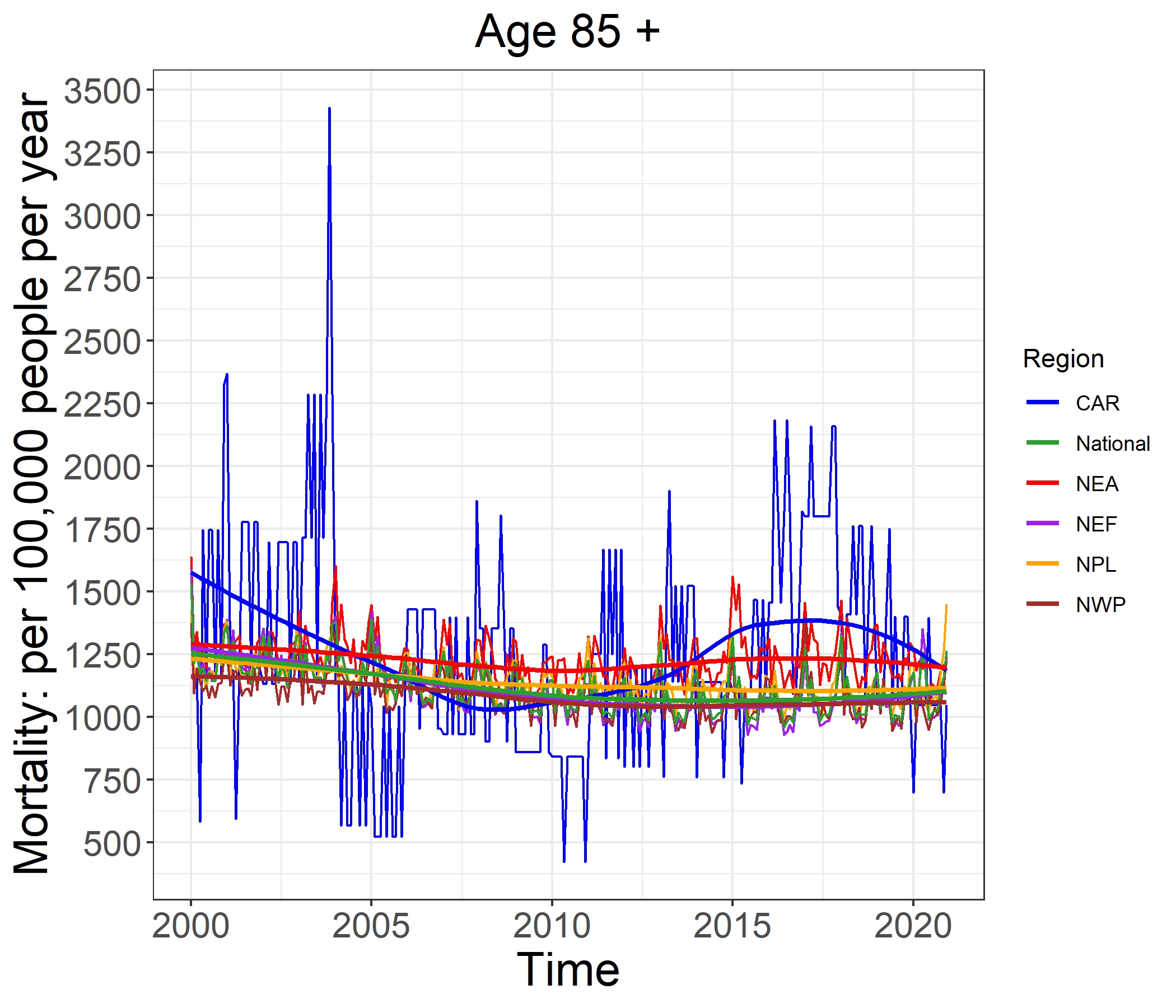

Supplement: Supplementary file 1 — Supplementary Material 1. [file 12889_2024_18785_MOESM1_ESM.zip › updated fig/mortality_85.jpeg]

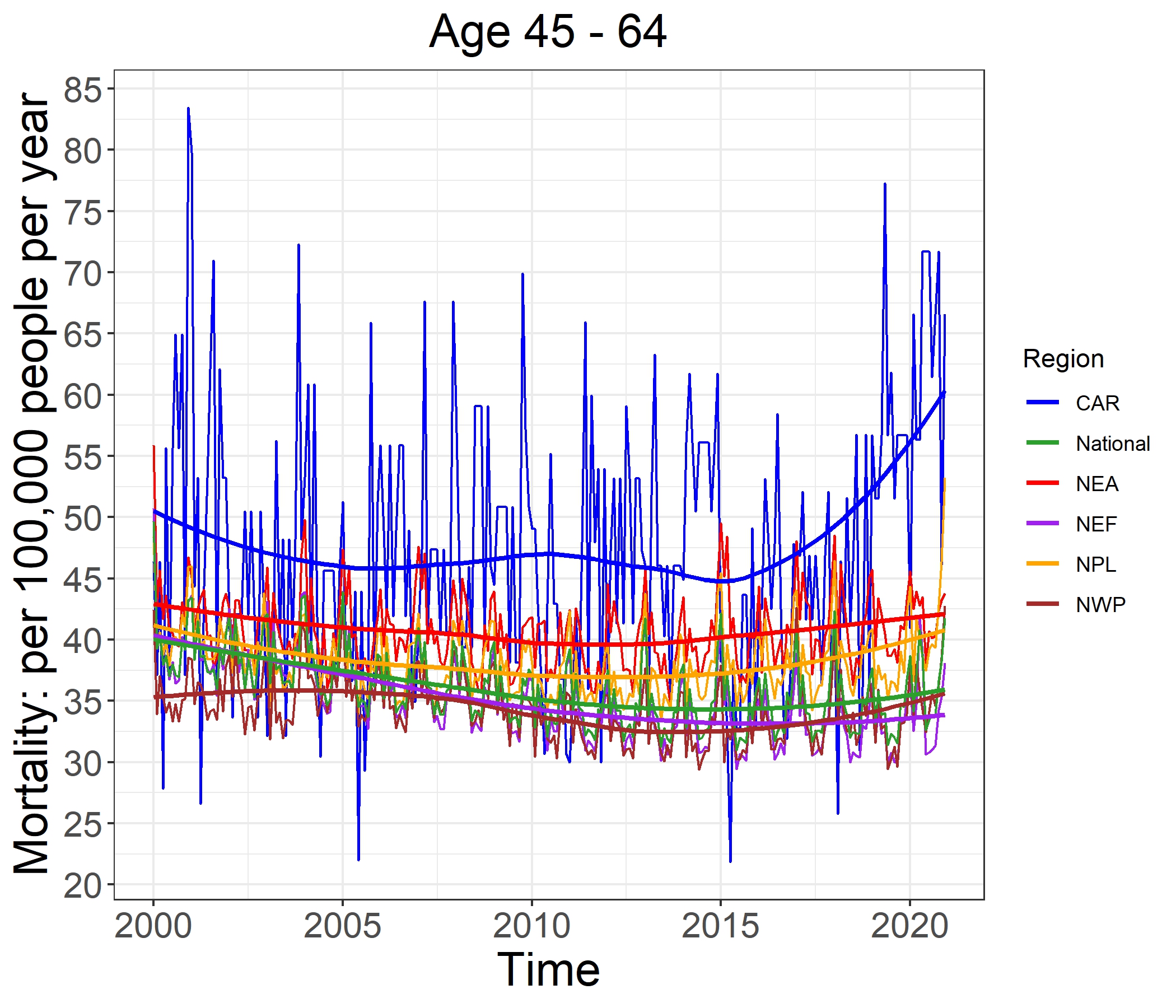

Supplement: Supplementary file 1 — Supplementary Material 1. [file 12889_2024_18785_MOESM1_ESM.zip › updated fig/mortality_4564.jpeg]

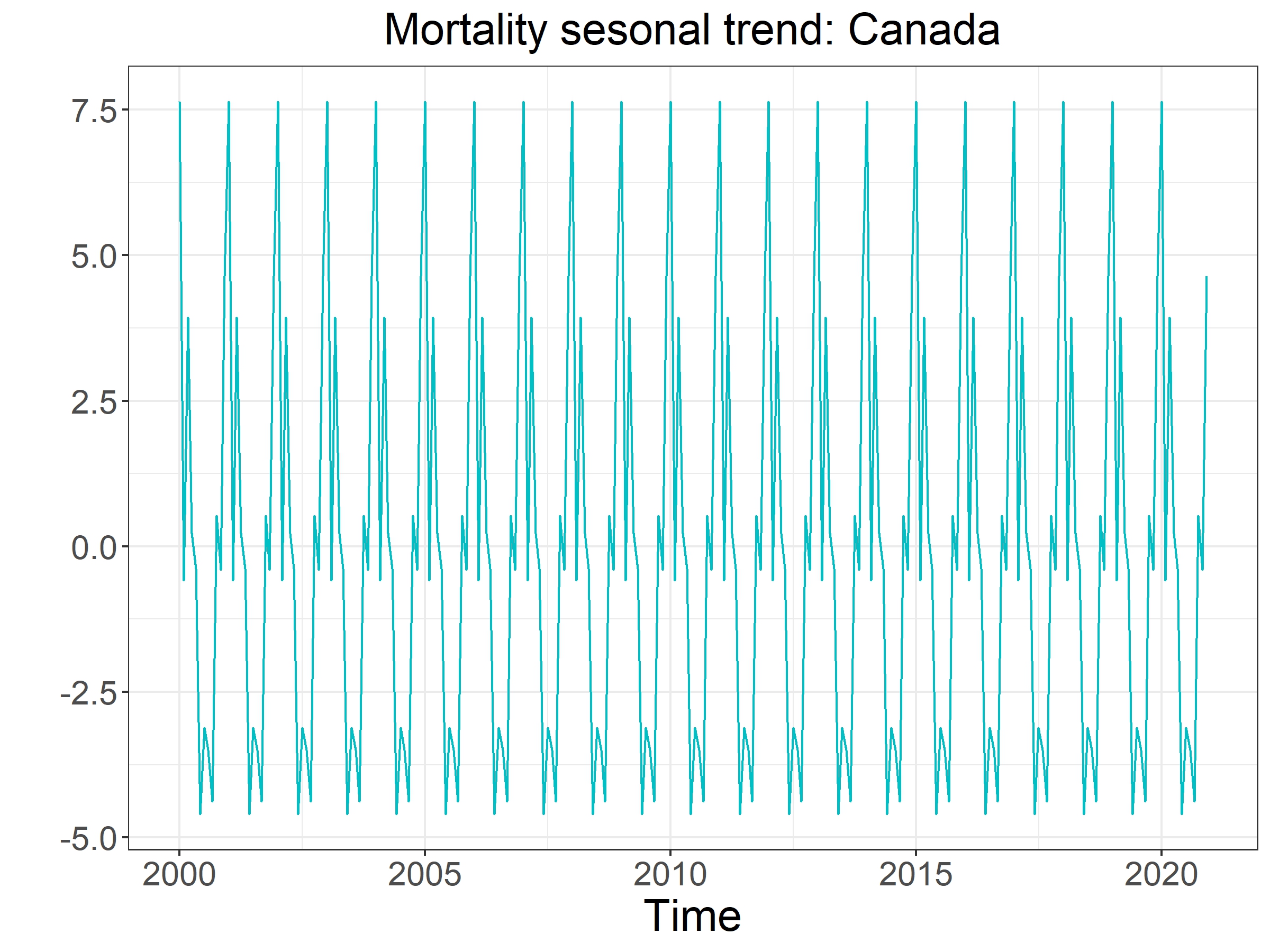

Supplement: Supplementary file 1 — Supplementary Material 1. [file 12889_2024_18785_MOESM1_ESM.zip › updated fig/Fig1_Canada_seasonal_trend.jpeg]

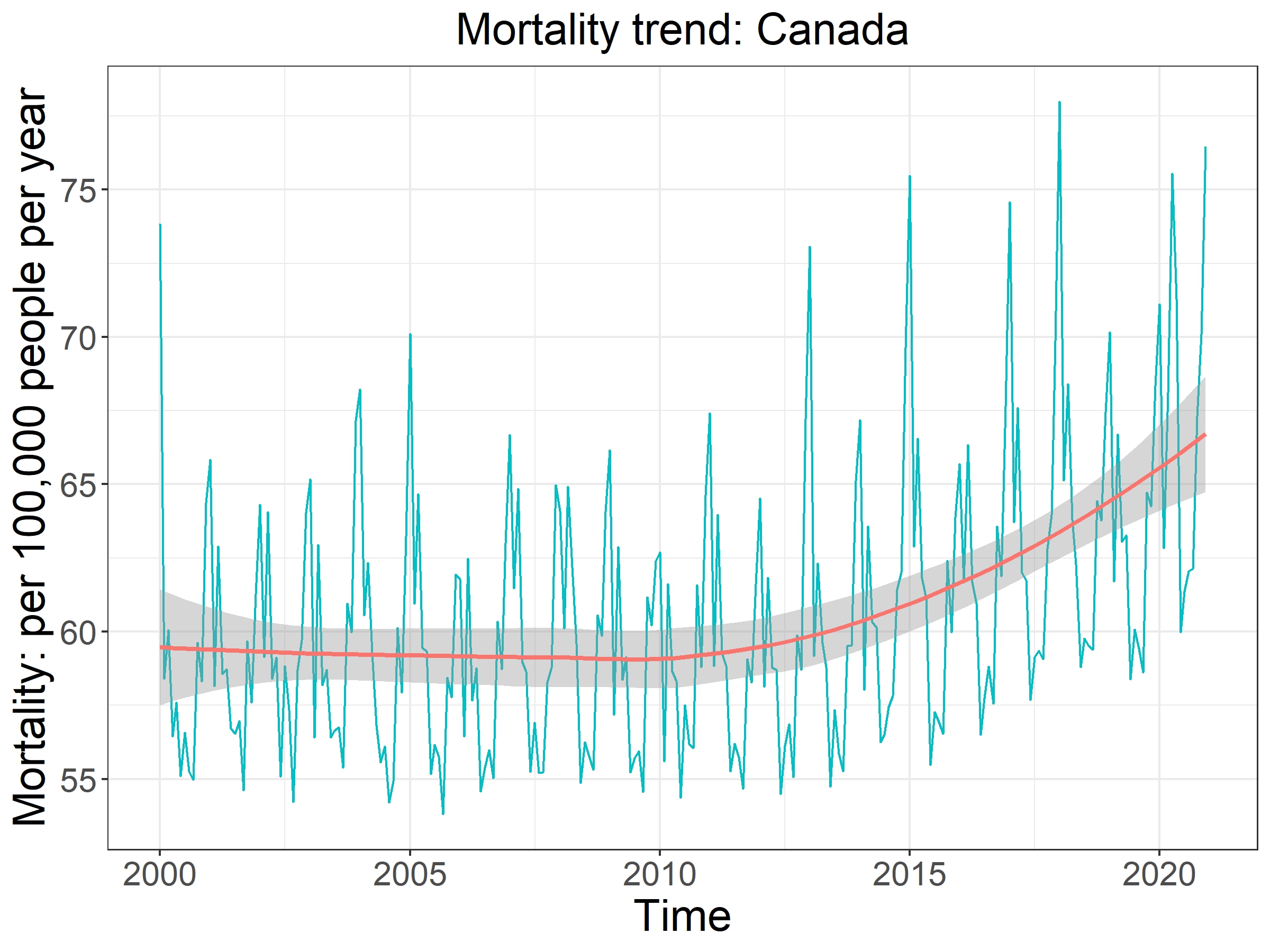

Supplement: Supplementary file 1 — Supplementary Material 1. [file 12889_2024_18785_MOESM1_ESM.zip › updated fig/Fig1_Canada_mortality_trend.jpeg]

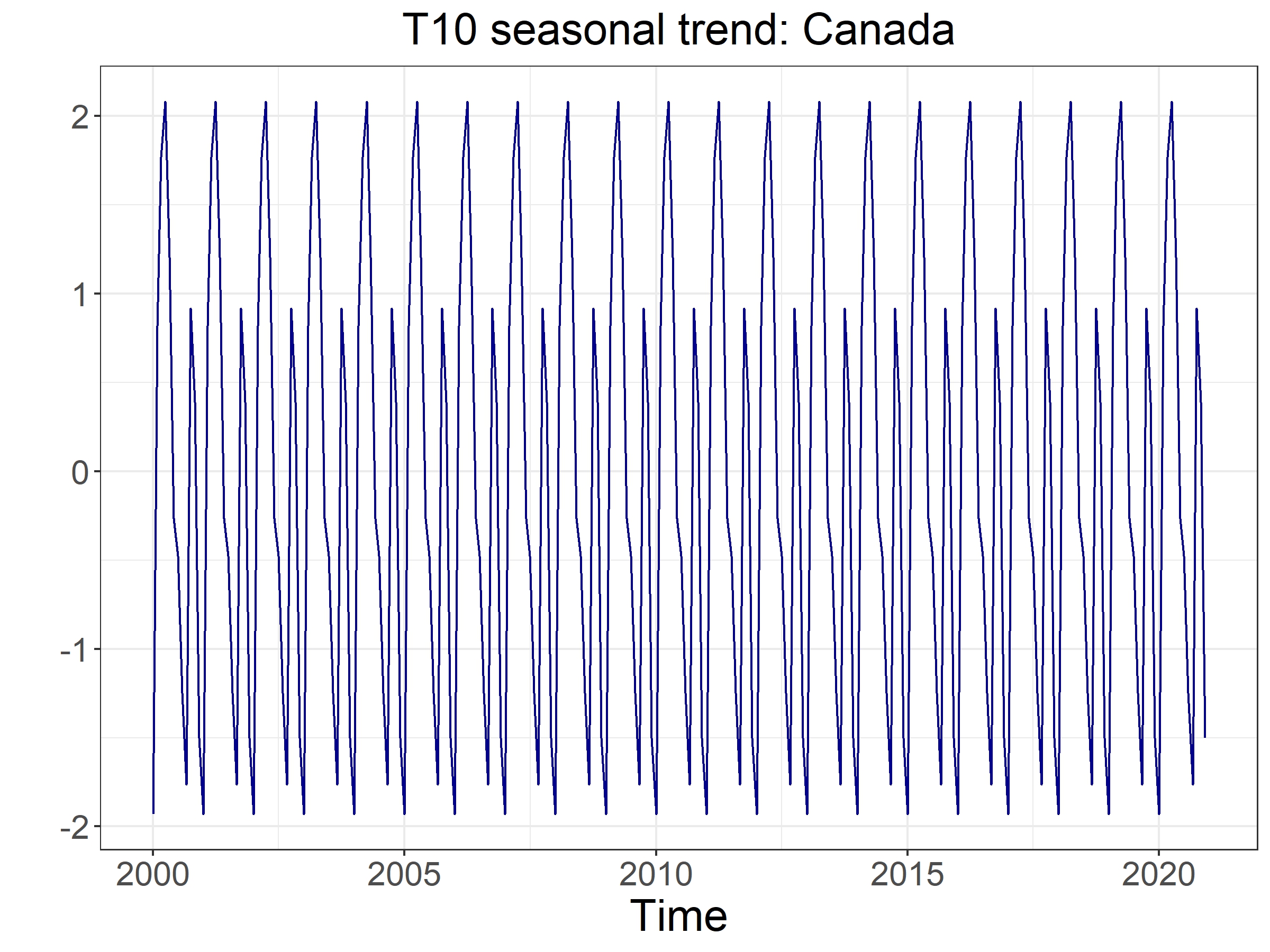

Supplement: Supplementary file 1 — Supplementary Material 1. [file 12889_2024_18785_MOESM1_ESM.zip › updated fig/Fig1_Canada_T10_seasonal_trend.jpeg]

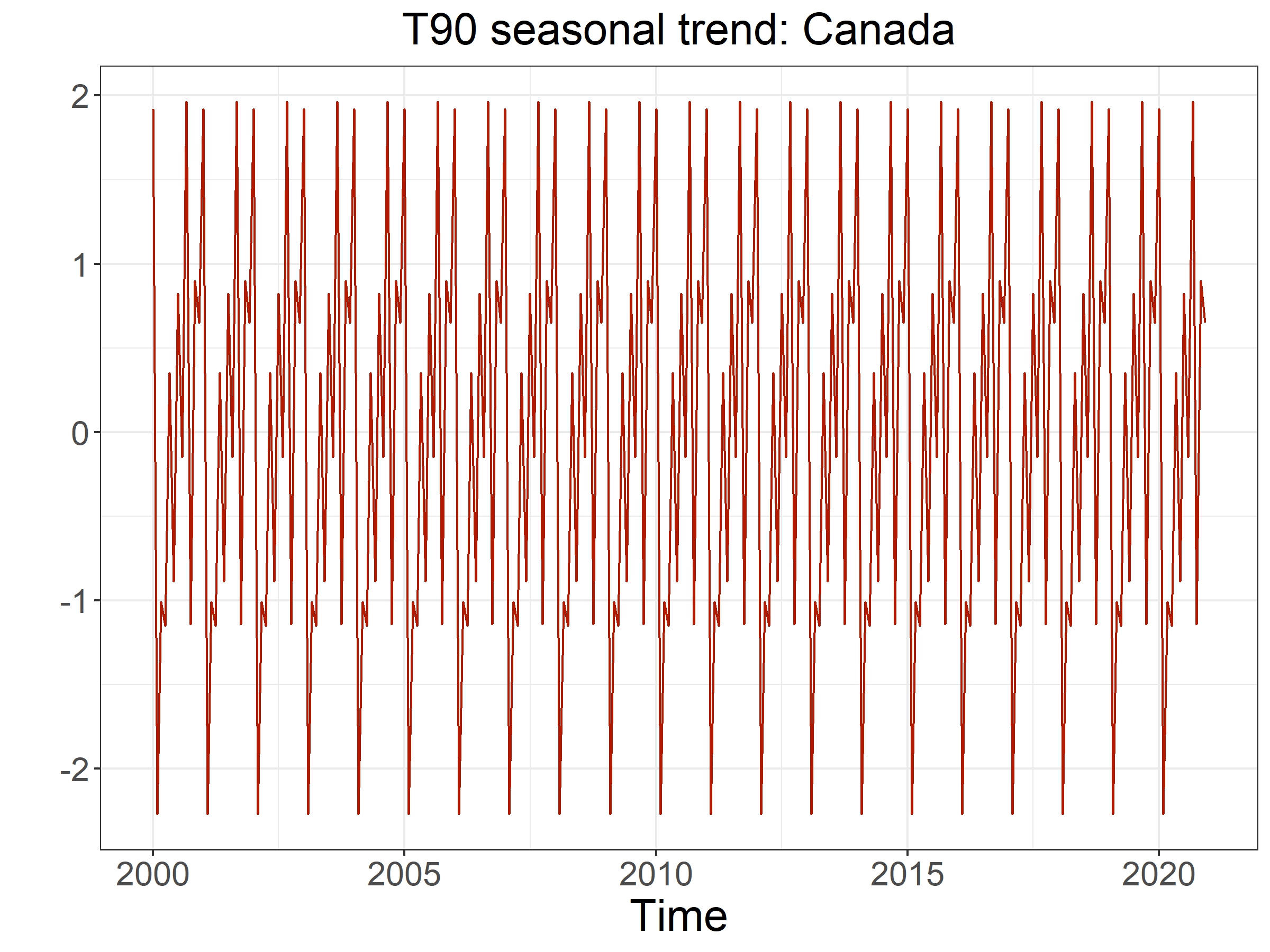

Supplement: Supplementary file 1 — Supplementary Material 1. [file 12889_2024_18785_MOESM1_ESM.zip › updated fig/Fig1_Canada_T90_seasonal_trend.jpeg]

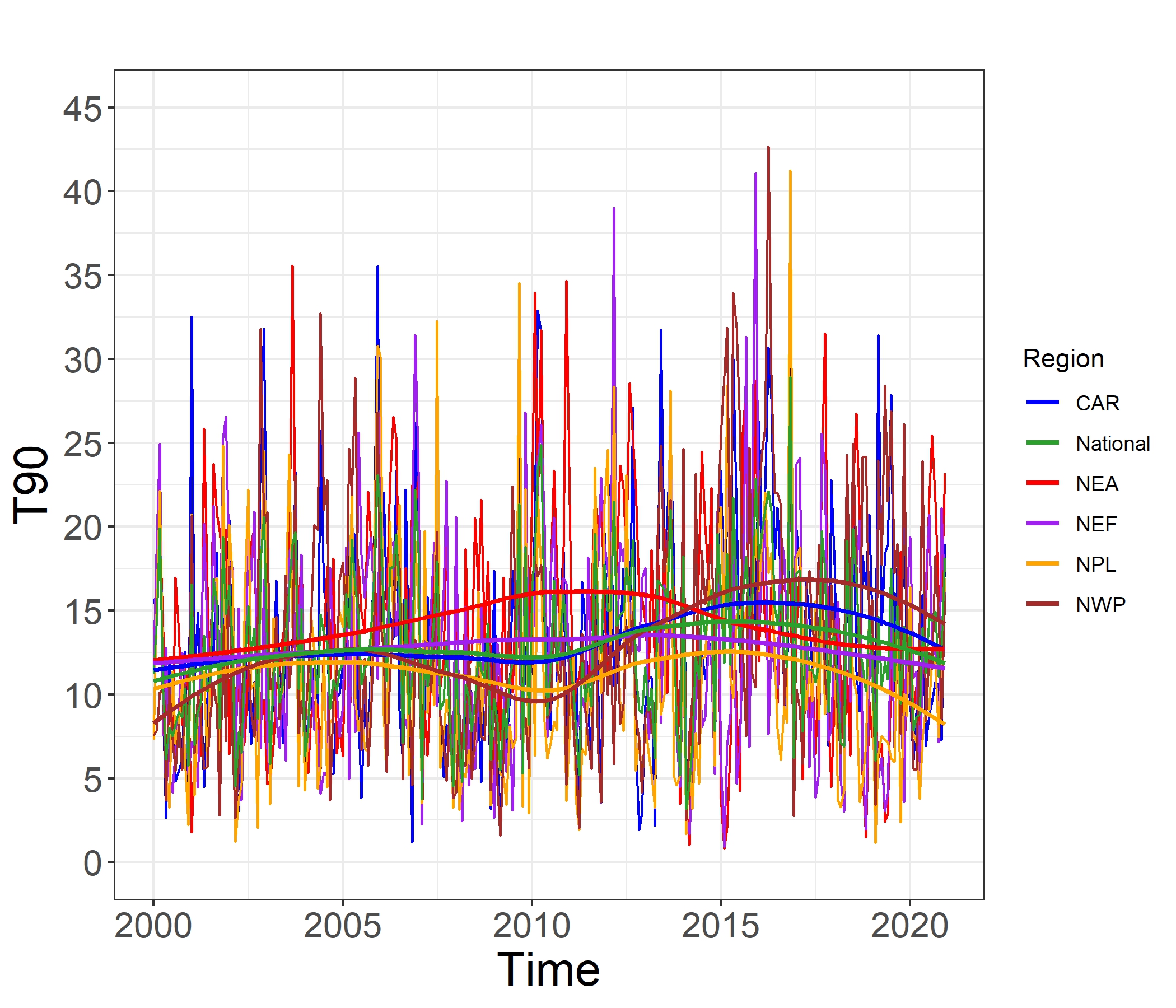

Supplement: Supplementary file 1 — Supplementary Material 1. [file 12889_2024_18785_MOESM1_ESM.zip › updated fig/temperature_plot_T90.jpeg]

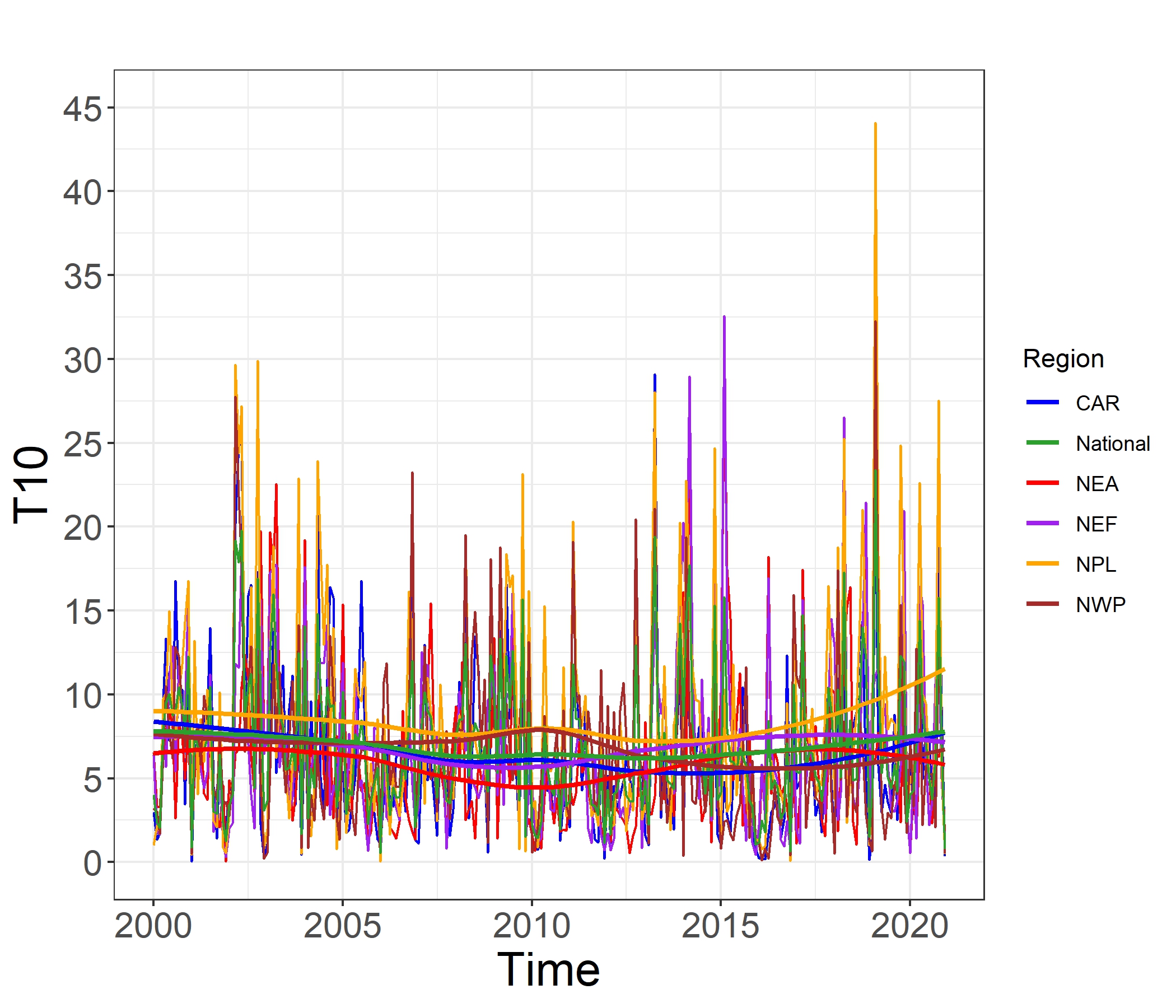

Supplement: Supplementary file 1 — Supplementary Material 1. [file 12889_2024_18785_MOESM1_ESM.zip › updated fig/temperature_plot_T10.jpeg]

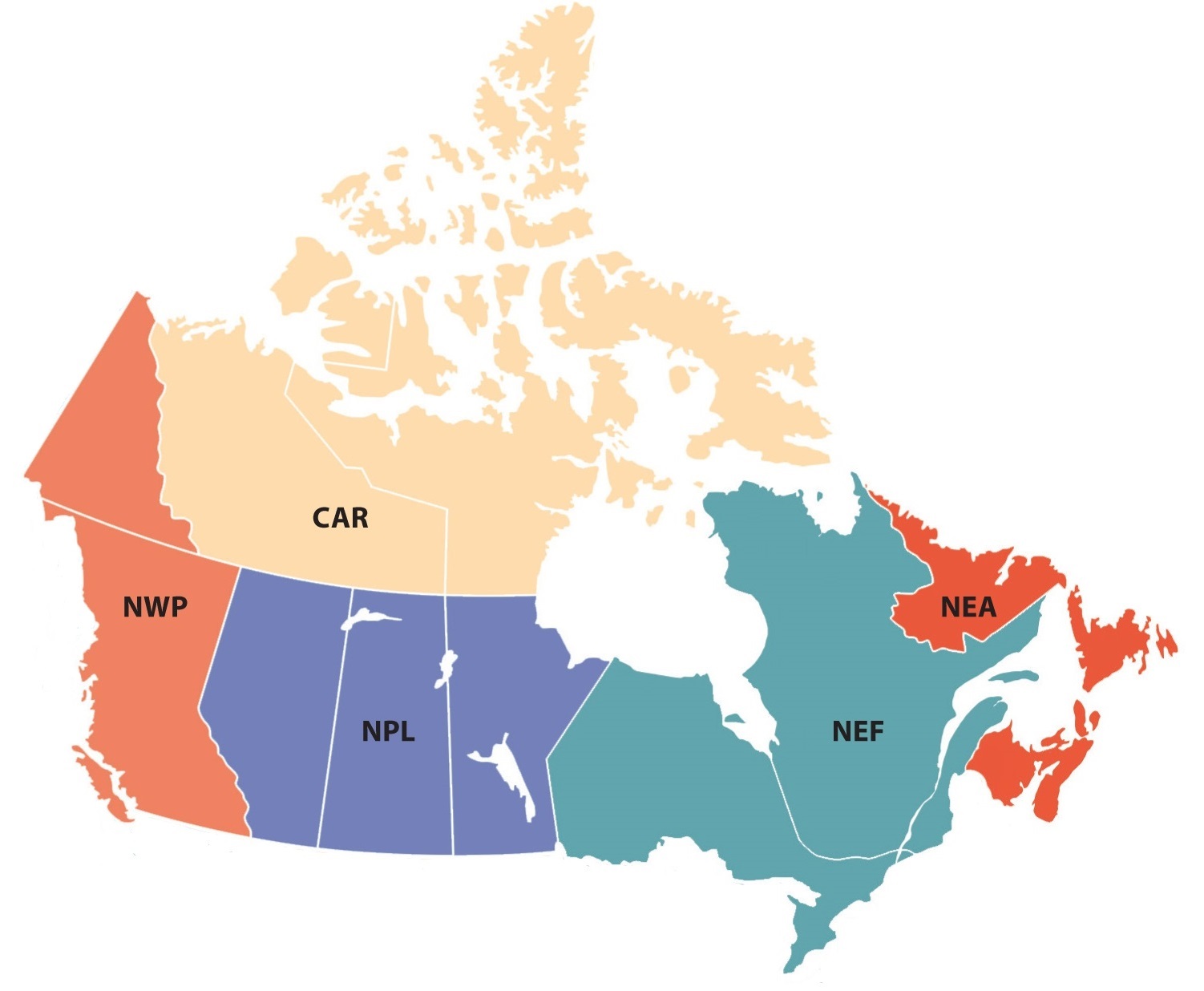

Supplement: Supplementary file 1 — Supplementary Material 1. [file 12889_2024_18785_MOESM1_ESM.zip › updated fig/regions-ACI.jpg]

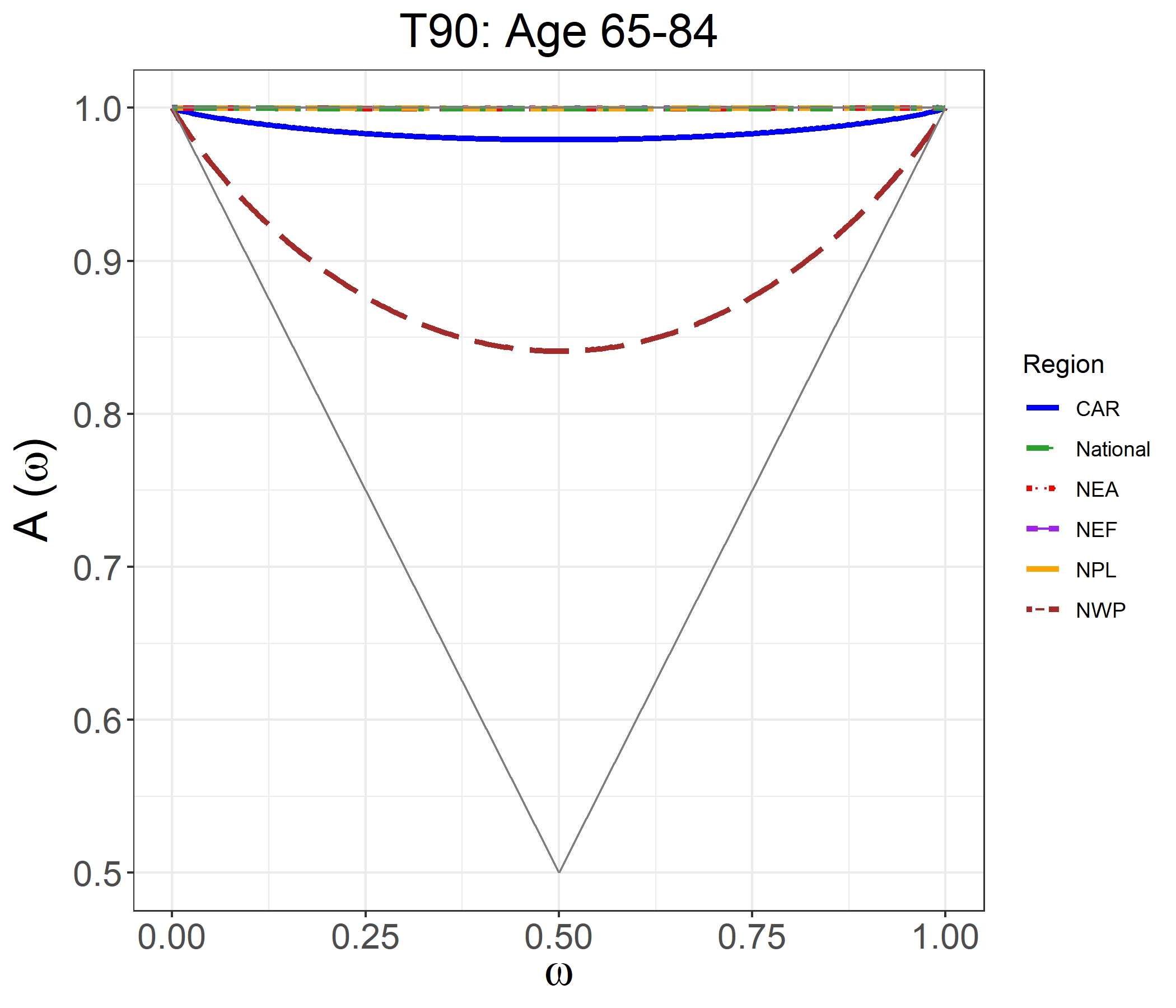

Supplement: Supplementary file 1 — Supplementary Material 1. [file 12889_2024_18785_MOESM1_ESM.zip › updated fig/T90_6584.jpeg]

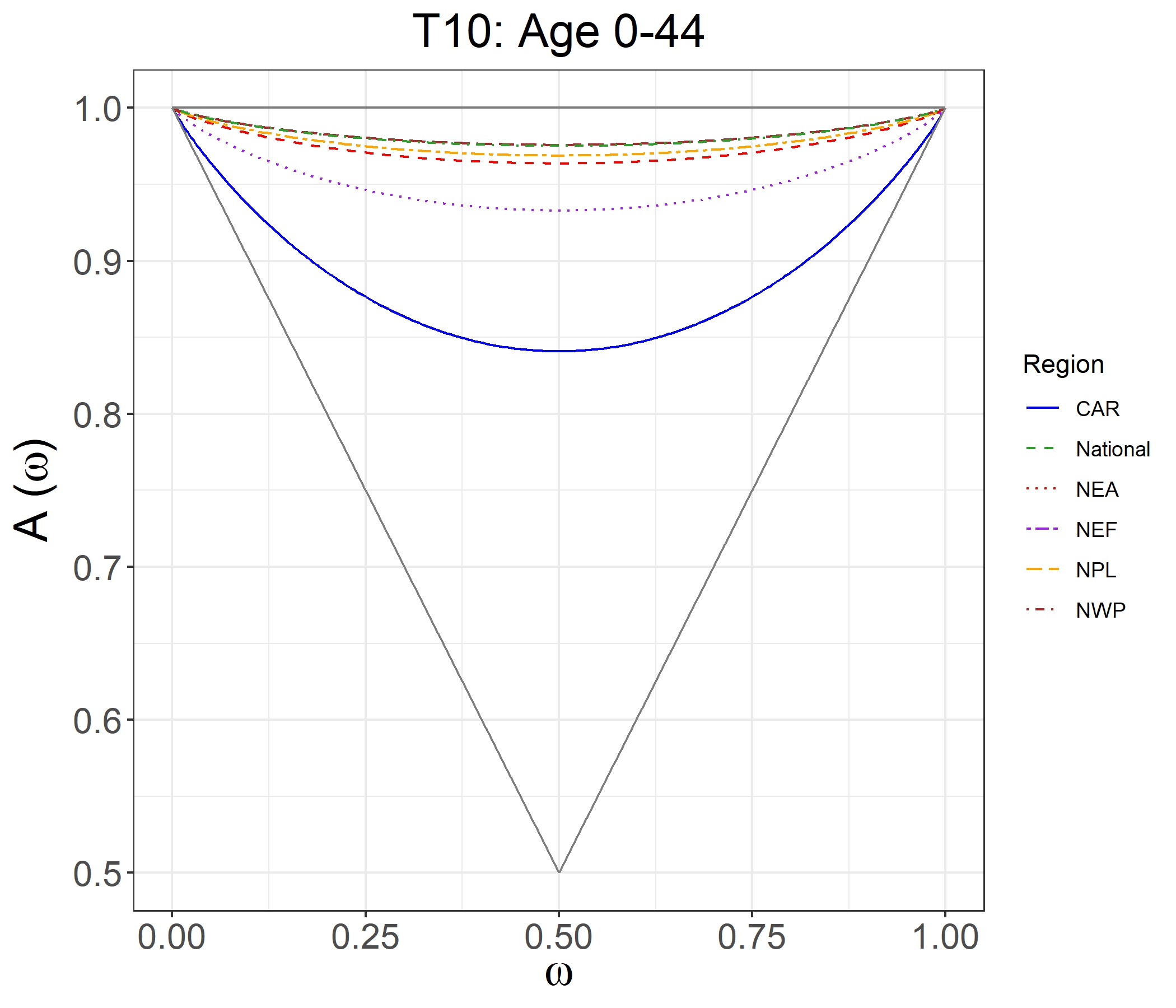

Supplement: Supplementary file 1 — Supplementary Material 1. [file 12889_2024_18785_MOESM1_ESM.zip › updated fig/T10_044.jpeg]

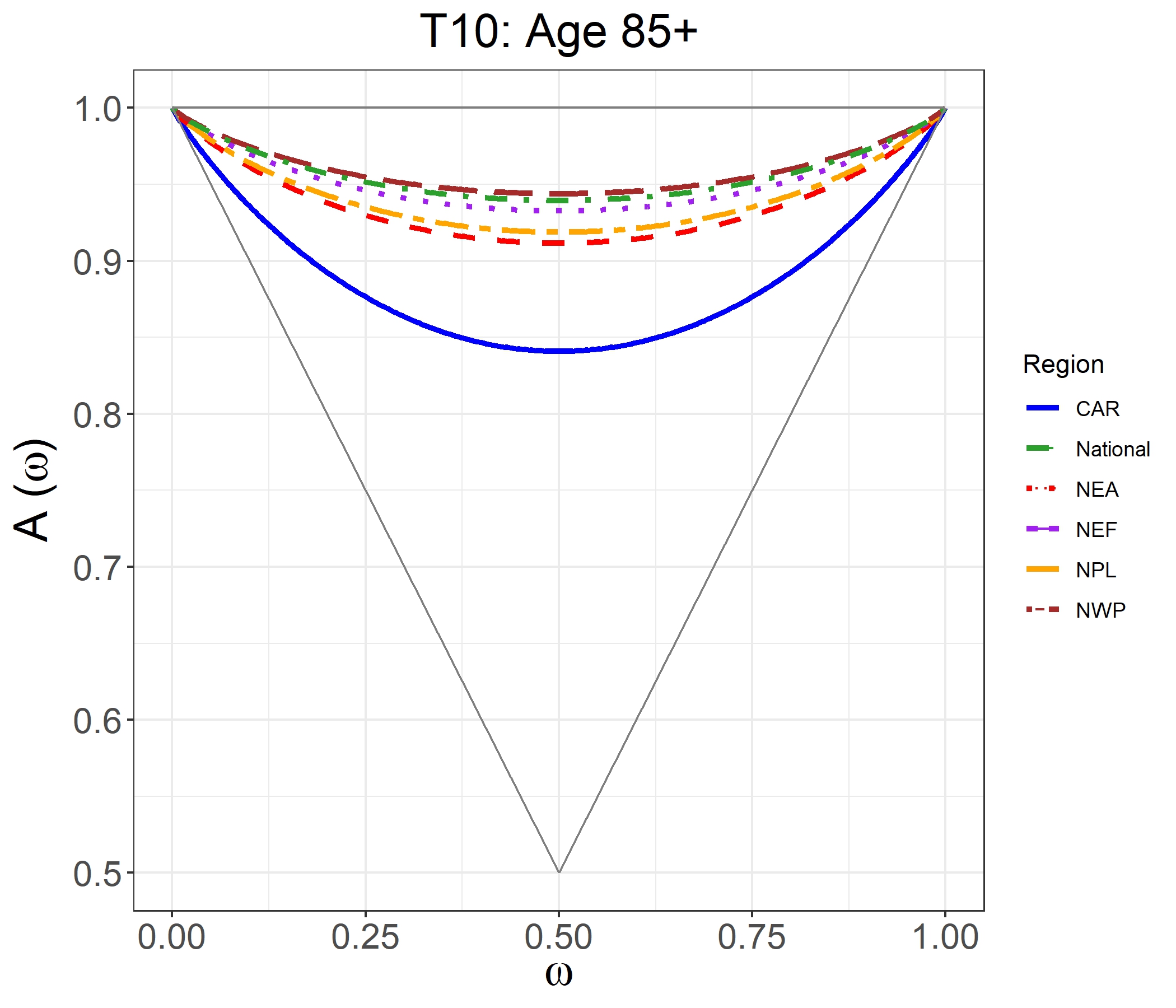

Supplement: Supplementary file 1 — Supplementary Material 1. [file 12889_2024_18785_MOESM1_ESM.zip › updated fig/T10_85.jpeg]

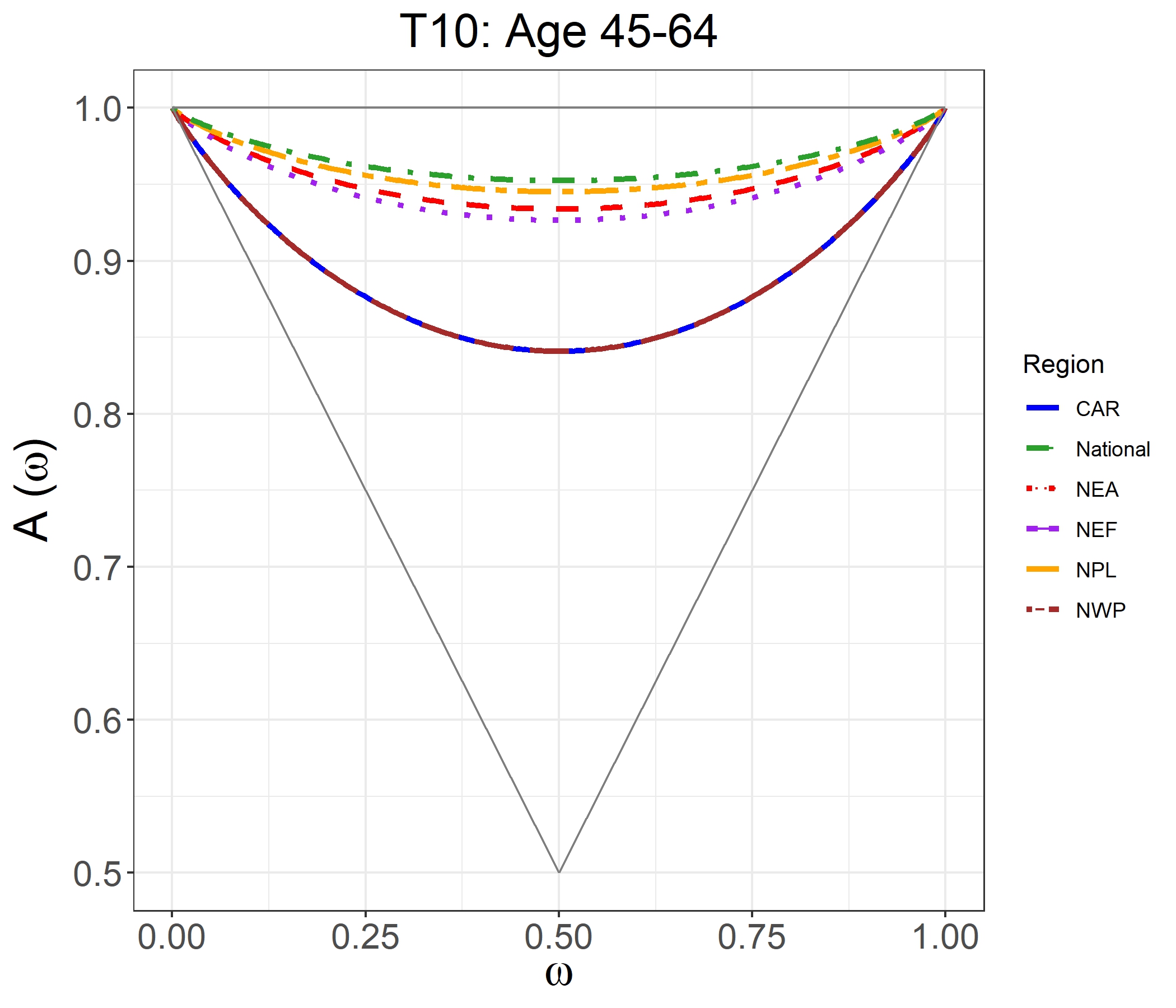

Supplement: Supplementary file 1 — Supplementary Material 1. [file 12889_2024_18785_MOESM1_ESM.zip › updated fig/T10_4564.jpeg]

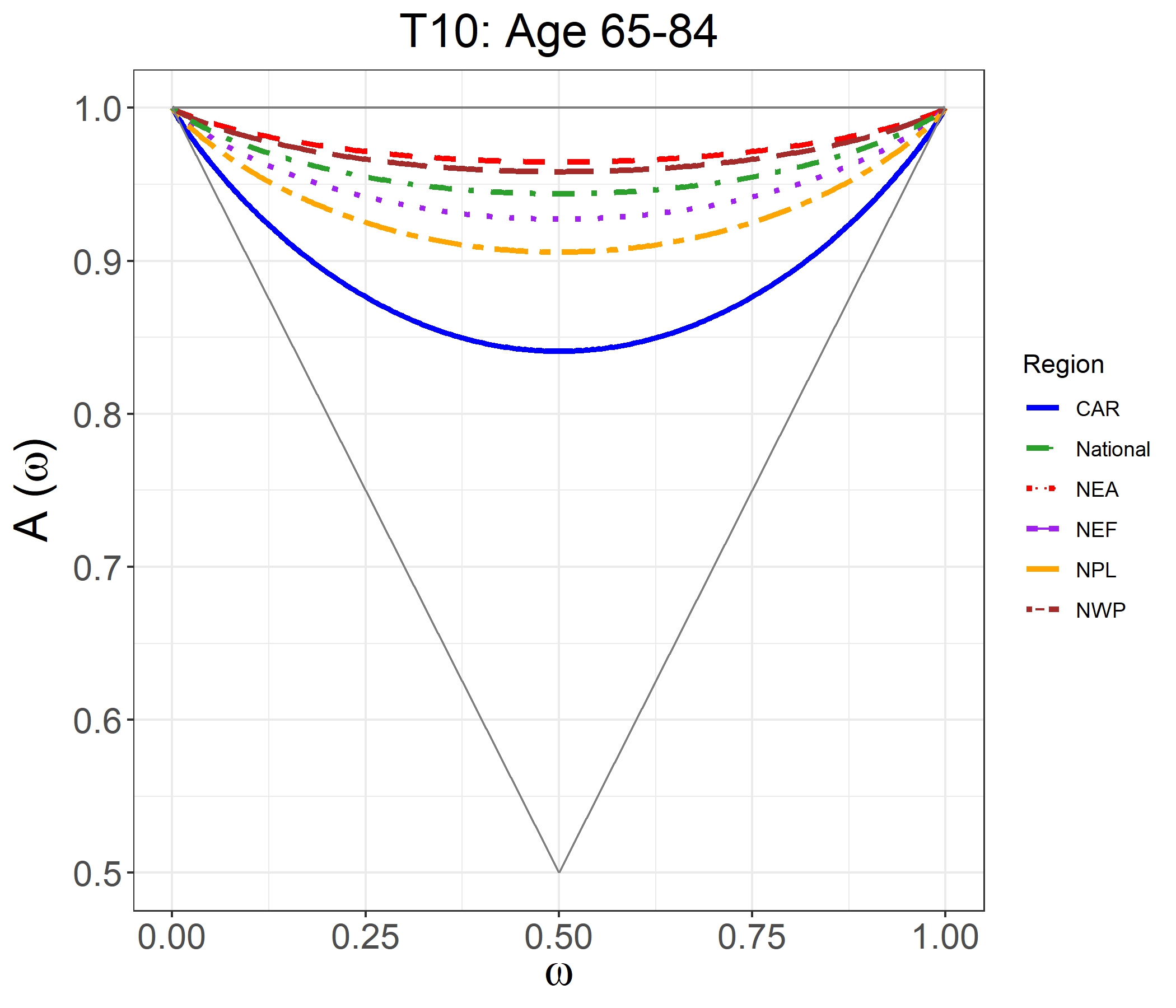

Supplement: Supplementary file 1 — Supplementary Material 1. [file 12889_2024_18785_MOESM1_ESM.zip › updated fig/T10_6584.jpeg]

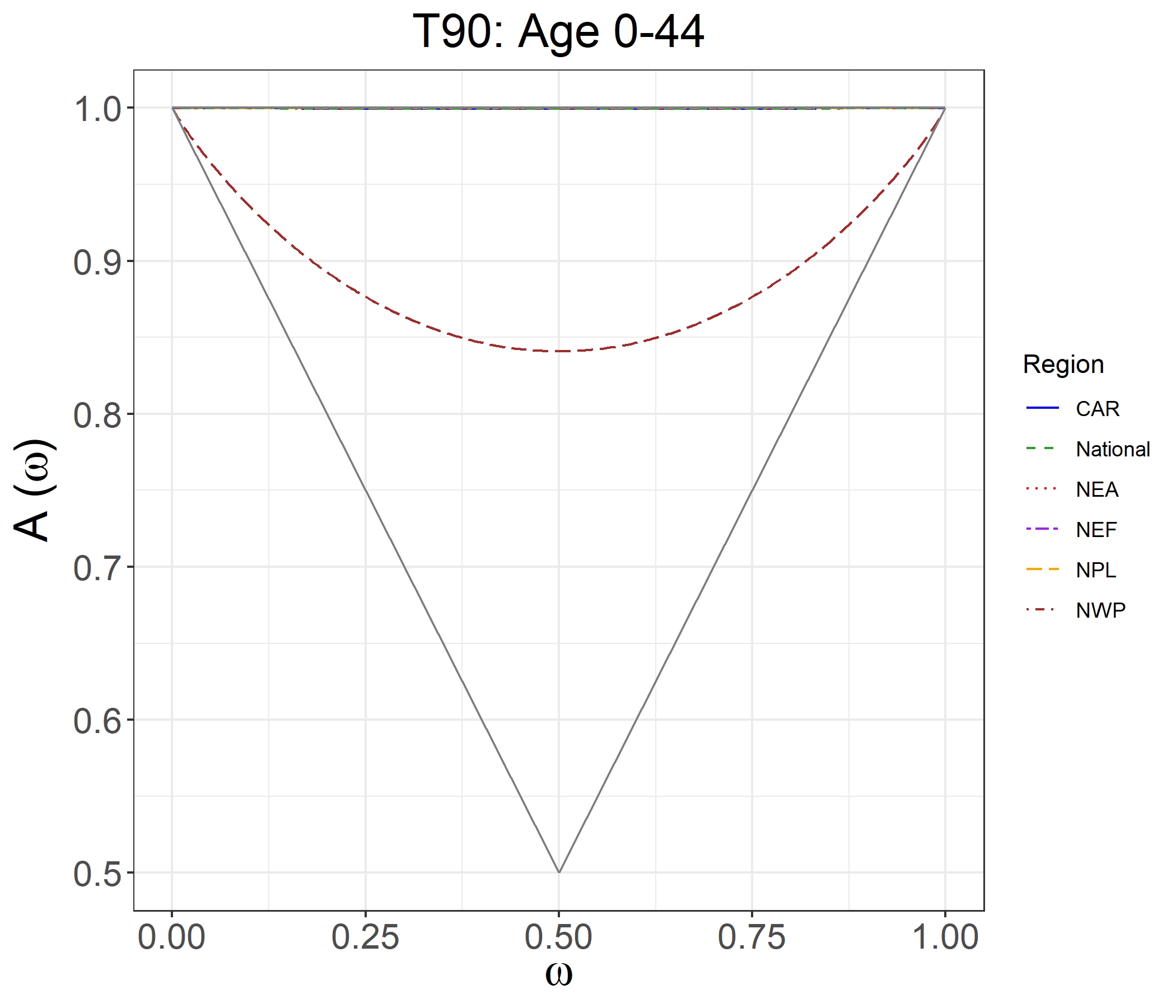

Supplement: Supplementary file 1 — Supplementary Material 1. [file 12889_2024_18785_MOESM1_ESM.zip › updated fig/T90_044.jpeg]

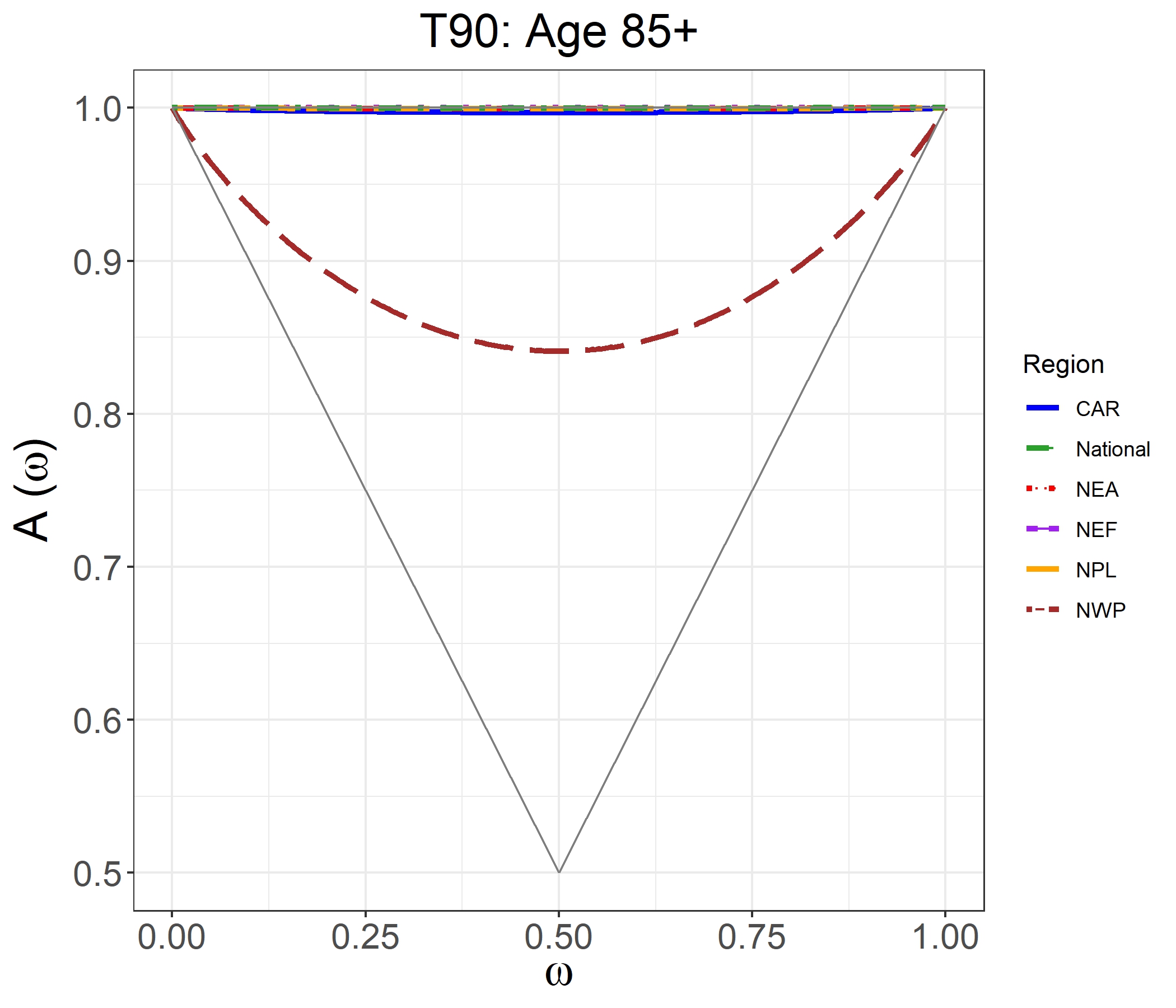

Supplement: Supplementary file 1 — Supplementary Material 1. [file 12889_2024_18785_MOESM1_ESM.zip › updated fig/T90_85.jpeg]

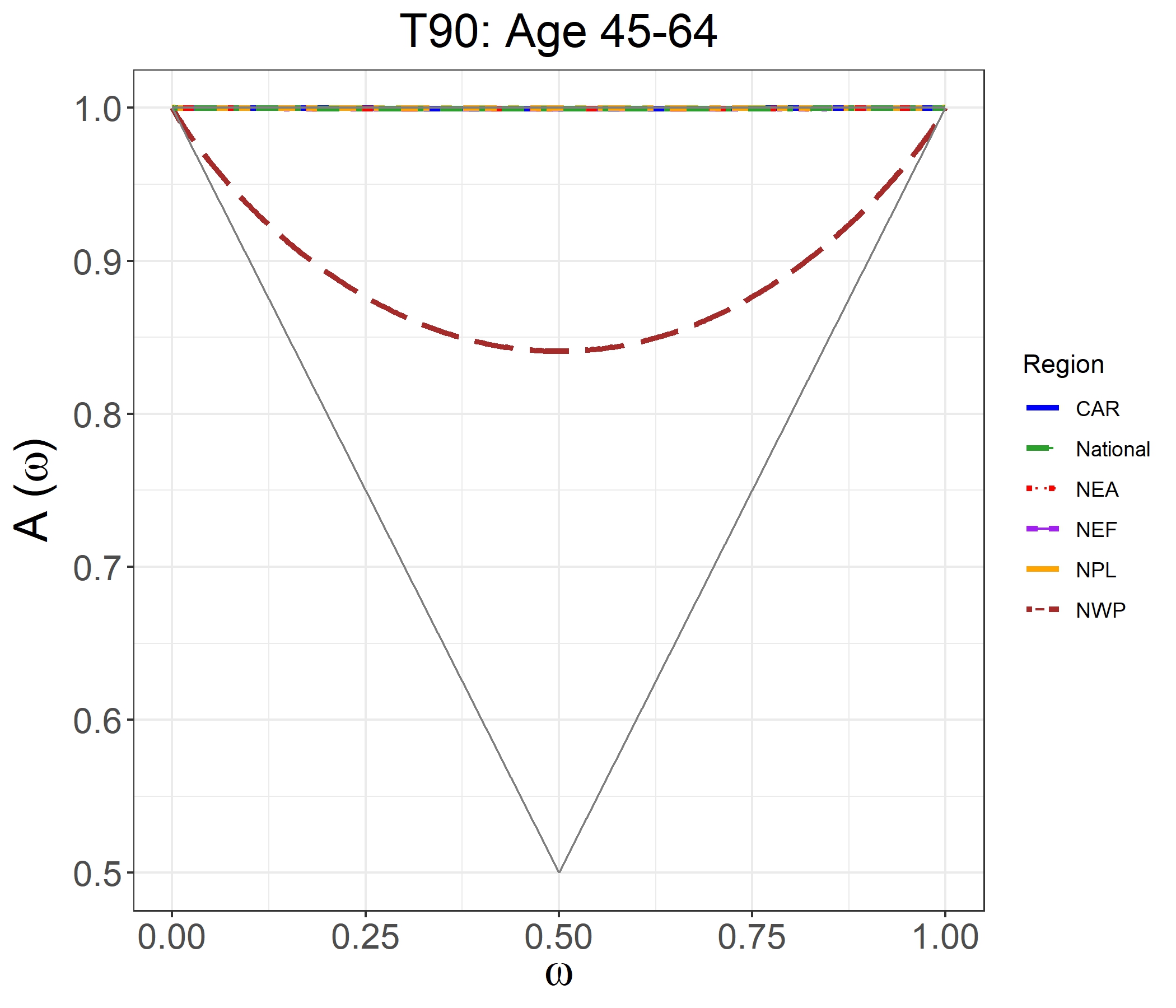

Supplement: Supplementary file 1 — Supplementary Material 1. [file 12889_2024_18785_MOESM1_ESM.zip › updated fig/T90_4564.jpeg]

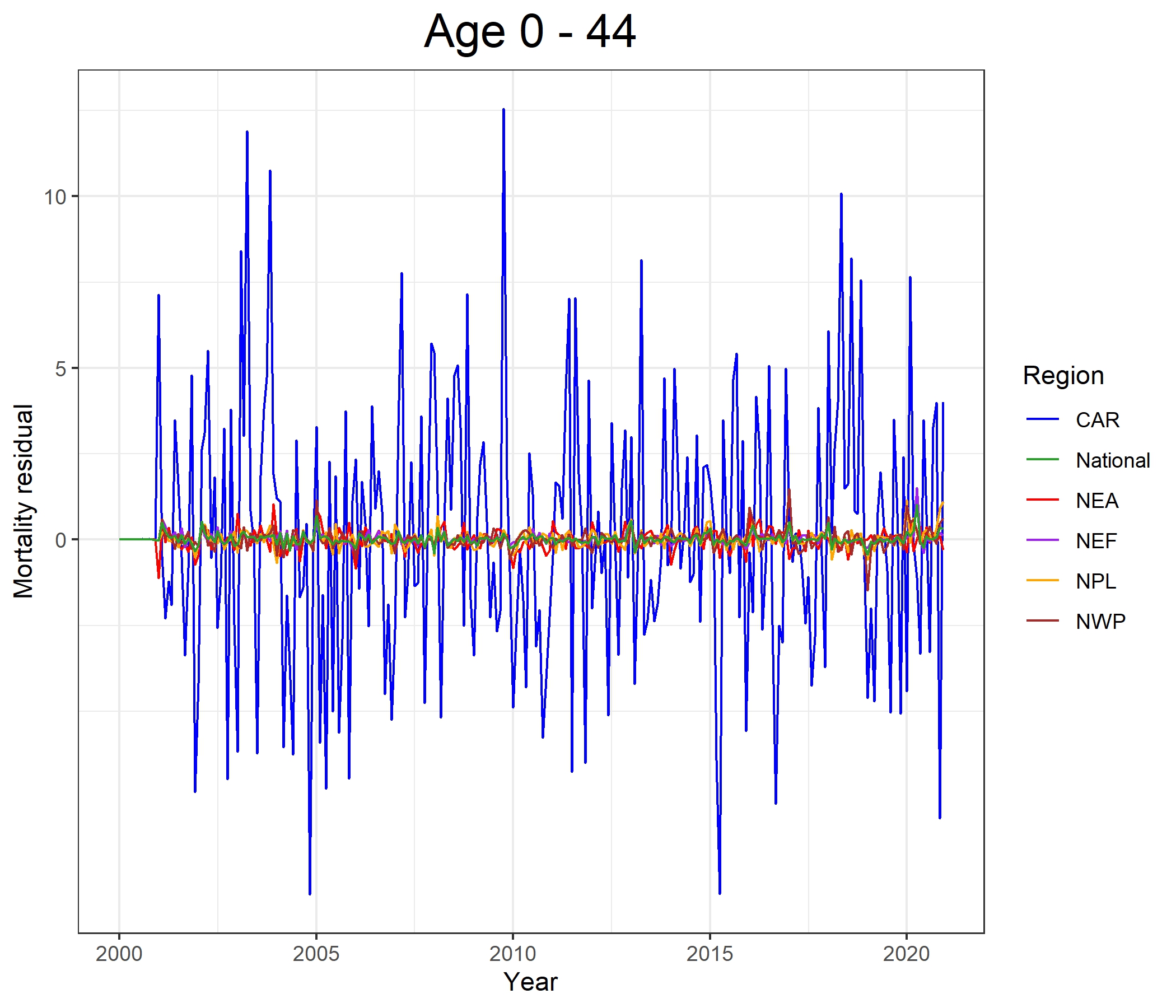

Supplement: Supplementary file 1 — Supplementary Material 1. [file 12889_2024_18785_MOESM1_ESM.zip › updated fig/mort_res_044.jpeg]

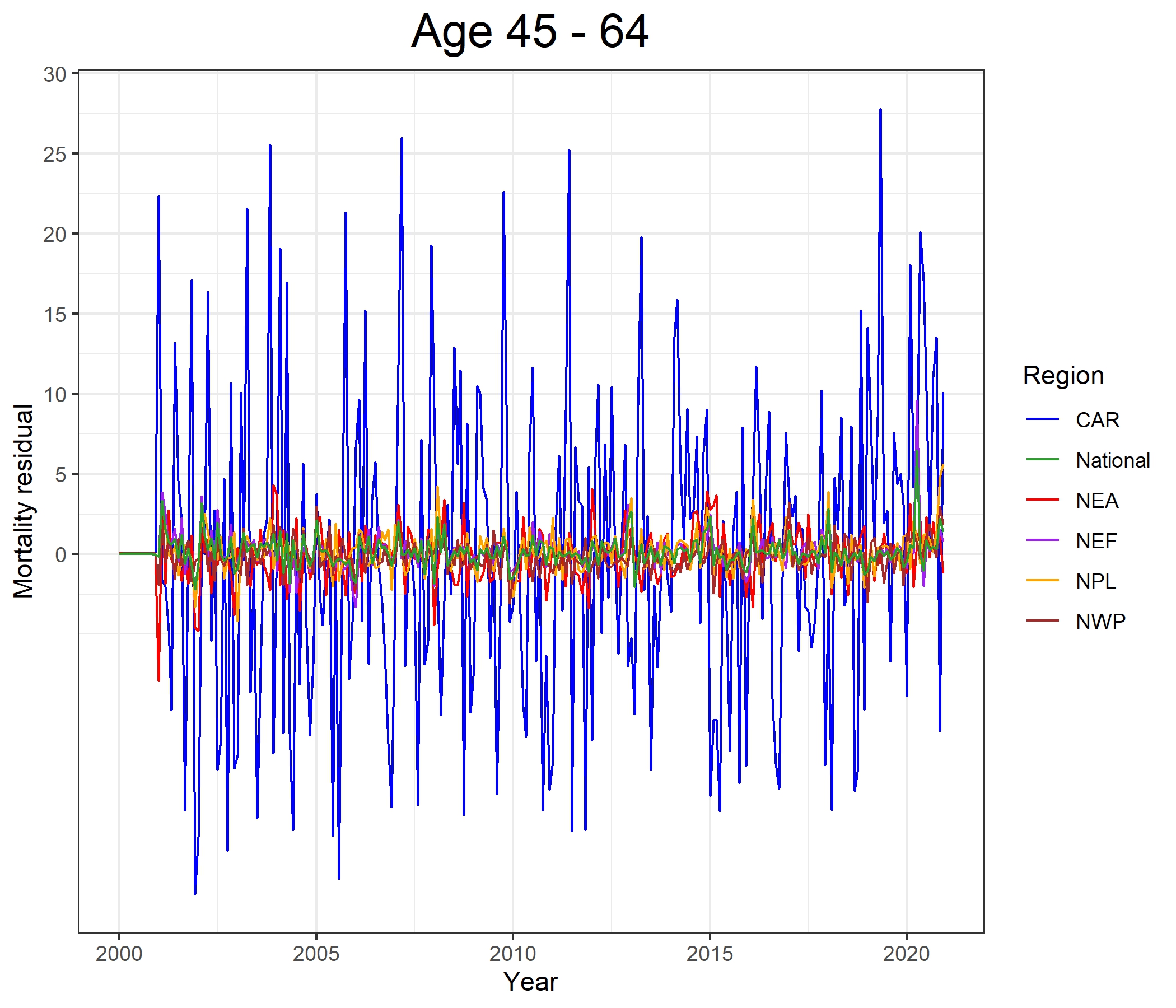

Supplement: Supplementary file 1 — Supplementary Material 1. [file 12889_2024_18785_MOESM1_ESM.zip › updated fig/mort_res_4564.jpeg]

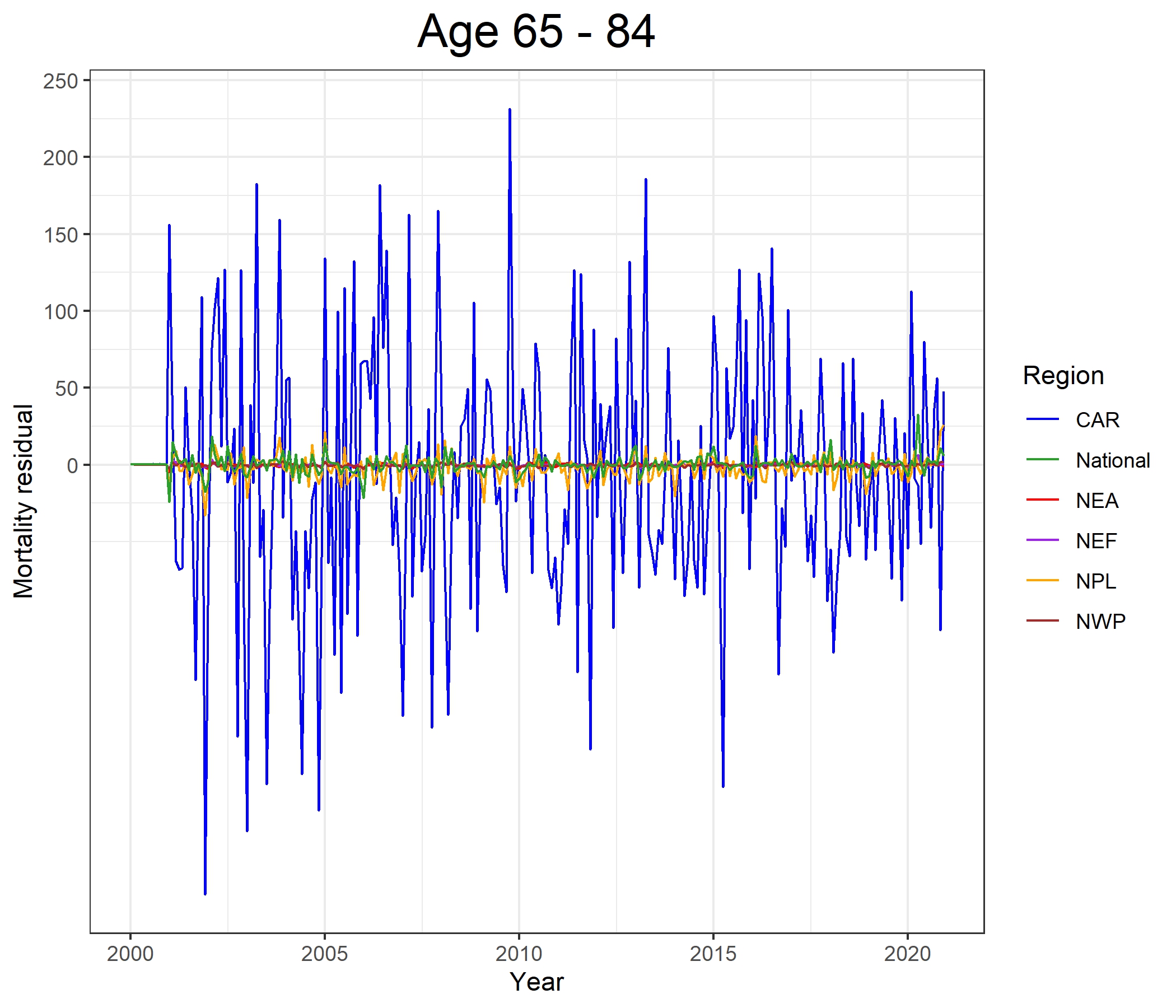

Supplement: Supplementary file 1 — Supplementary Material 1. [file 12889_2024_18785_MOESM1_ESM.zip › updated fig/mort_res_6584.jpeg]

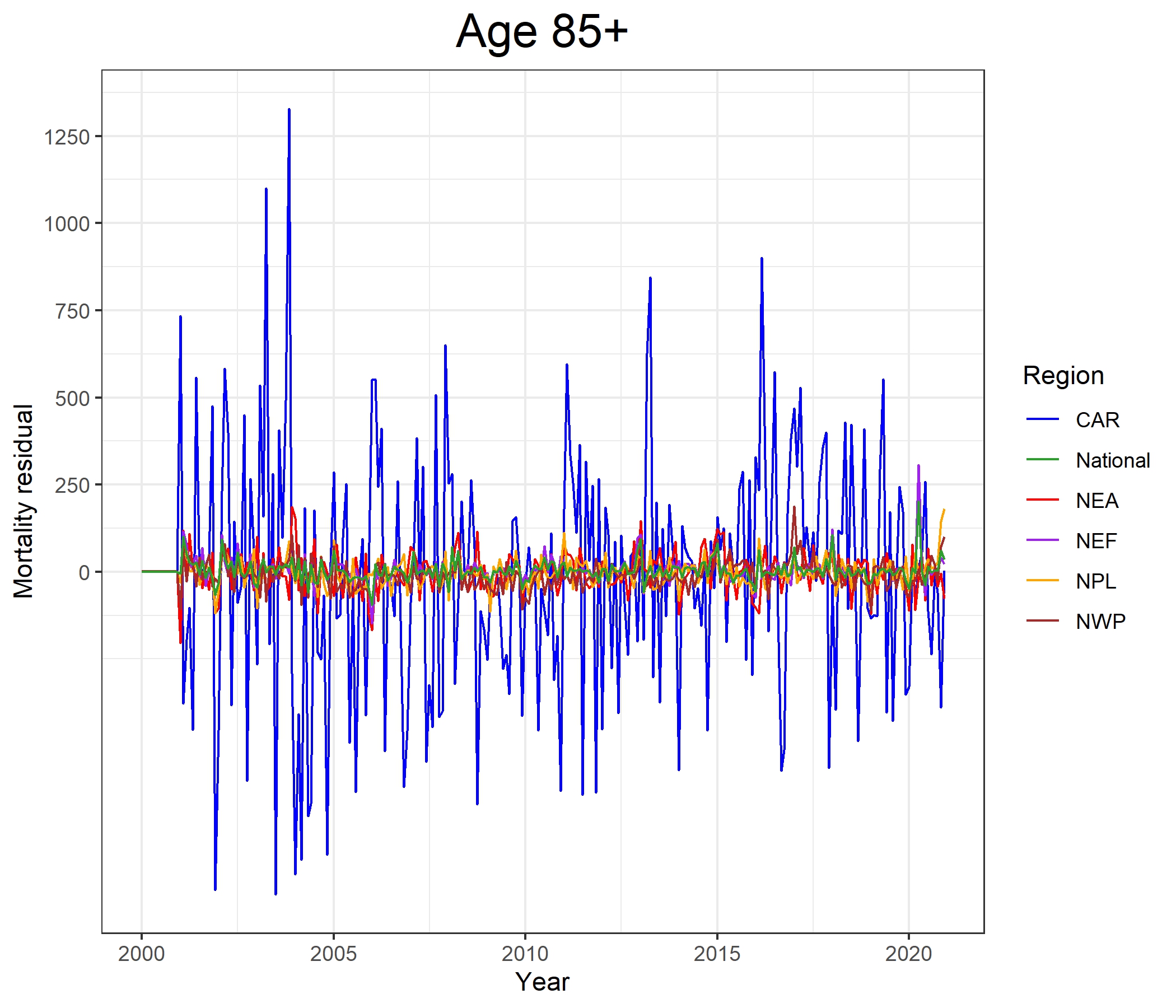

Supplement: Supplementary file 1 — Supplementary Material 1. [file 12889_2024_18785_MOESM1_ESM.zip › updated fig/mort_res_85.jpeg]

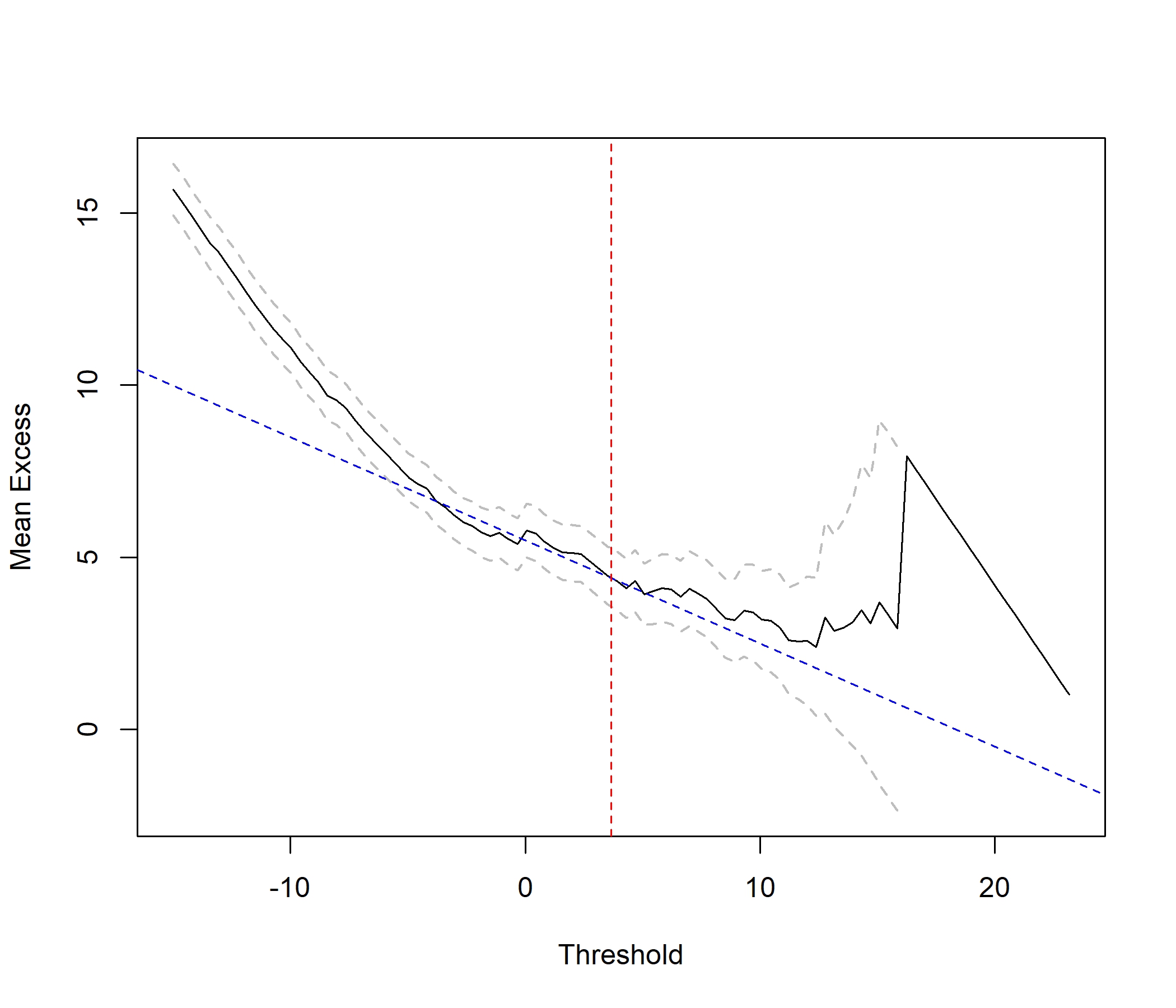

Supplement: Supplementary file 1 — Supplementary Material 1. [file 12889_2024_18785_MOESM1_ESM.zip › updated fig/mrl.jpeg]

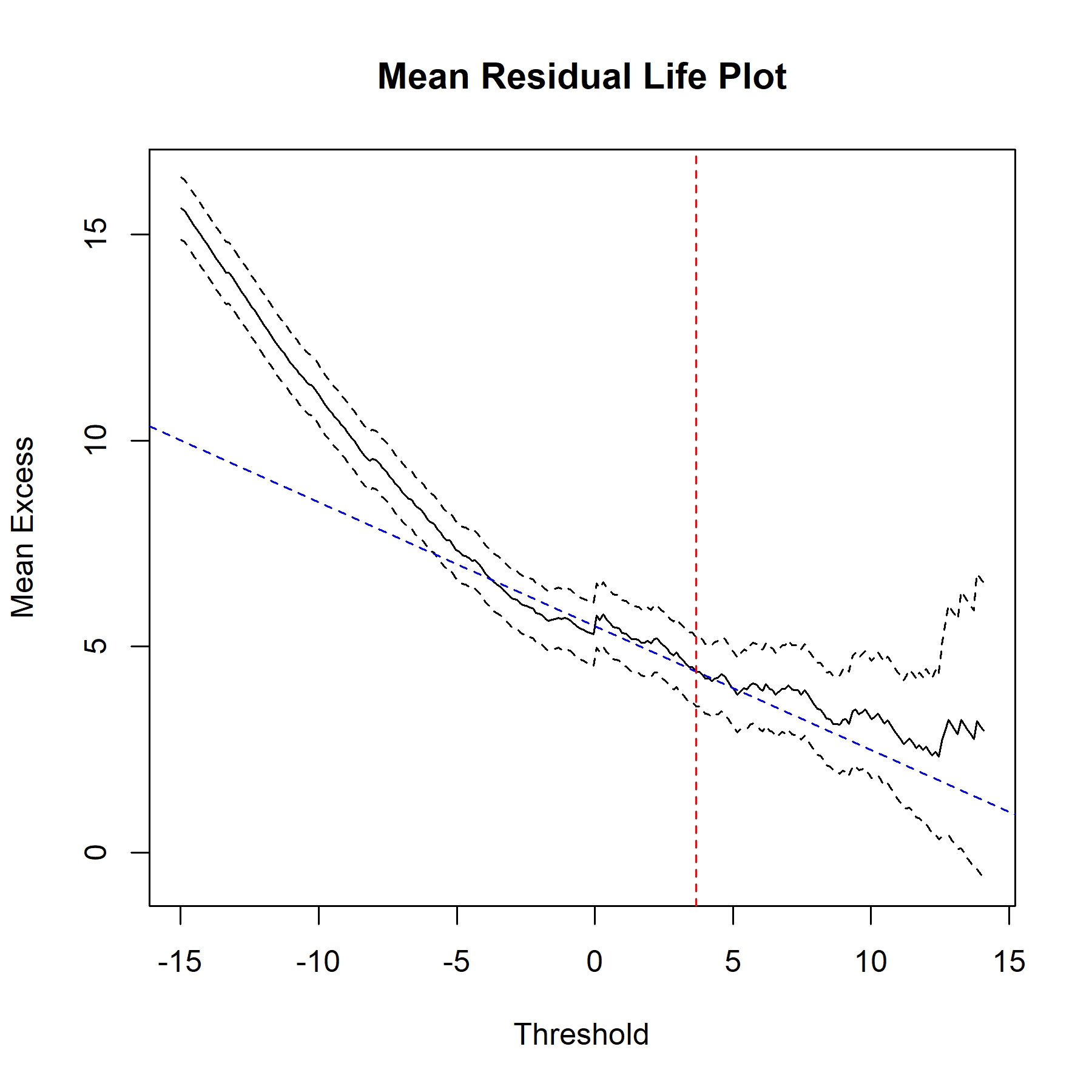

Supplement: Supplementary file 1 — Supplementary Material 1. [file 12889_2024_18785_MOESM1_ESM.zip › updated fig/mrl1.jpeg]

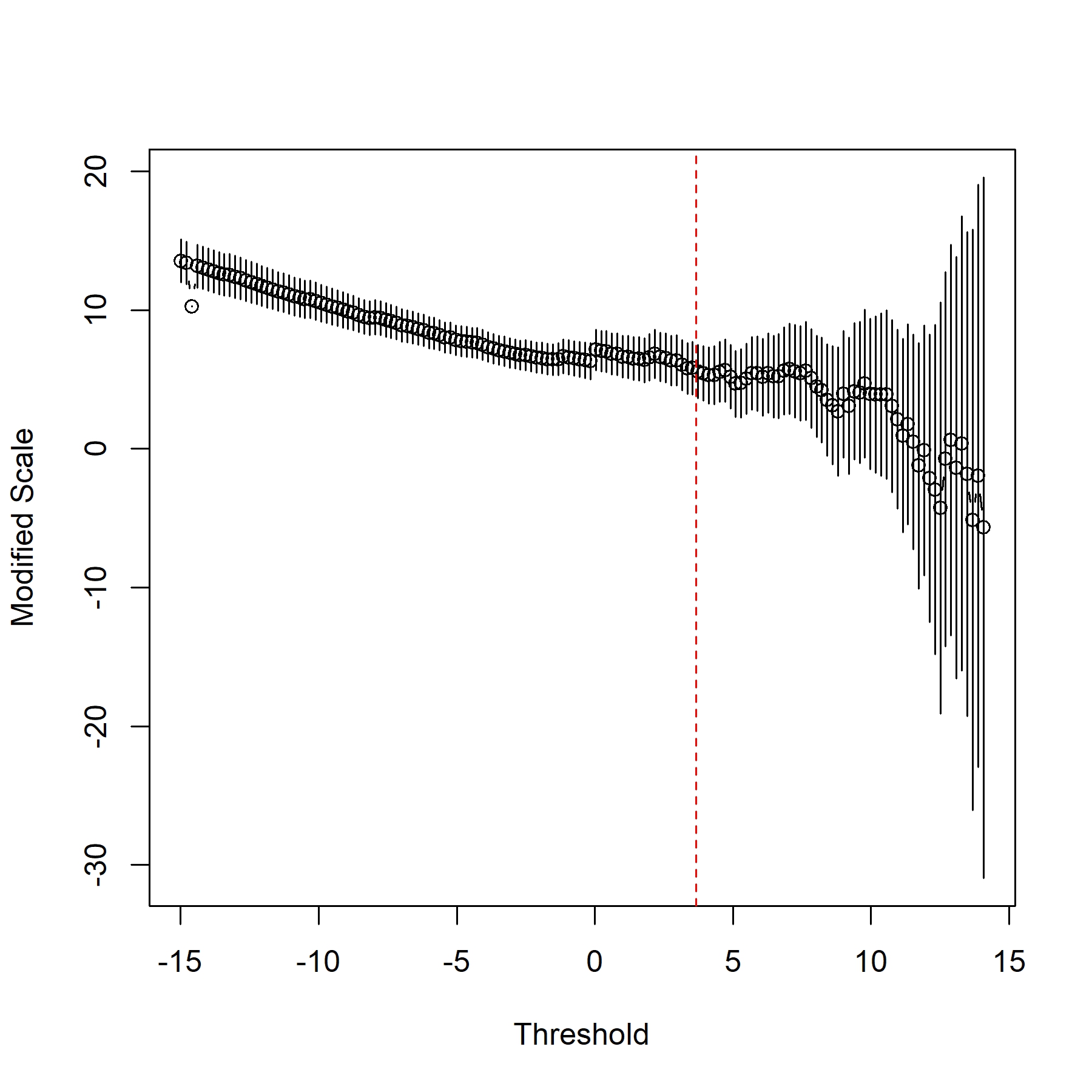

Supplement: Supplementary file 1 — Supplementary Material 1. [file 12889_2024_18785_MOESM1_ESM.zip › updated fig/stability_scale.jpeg]

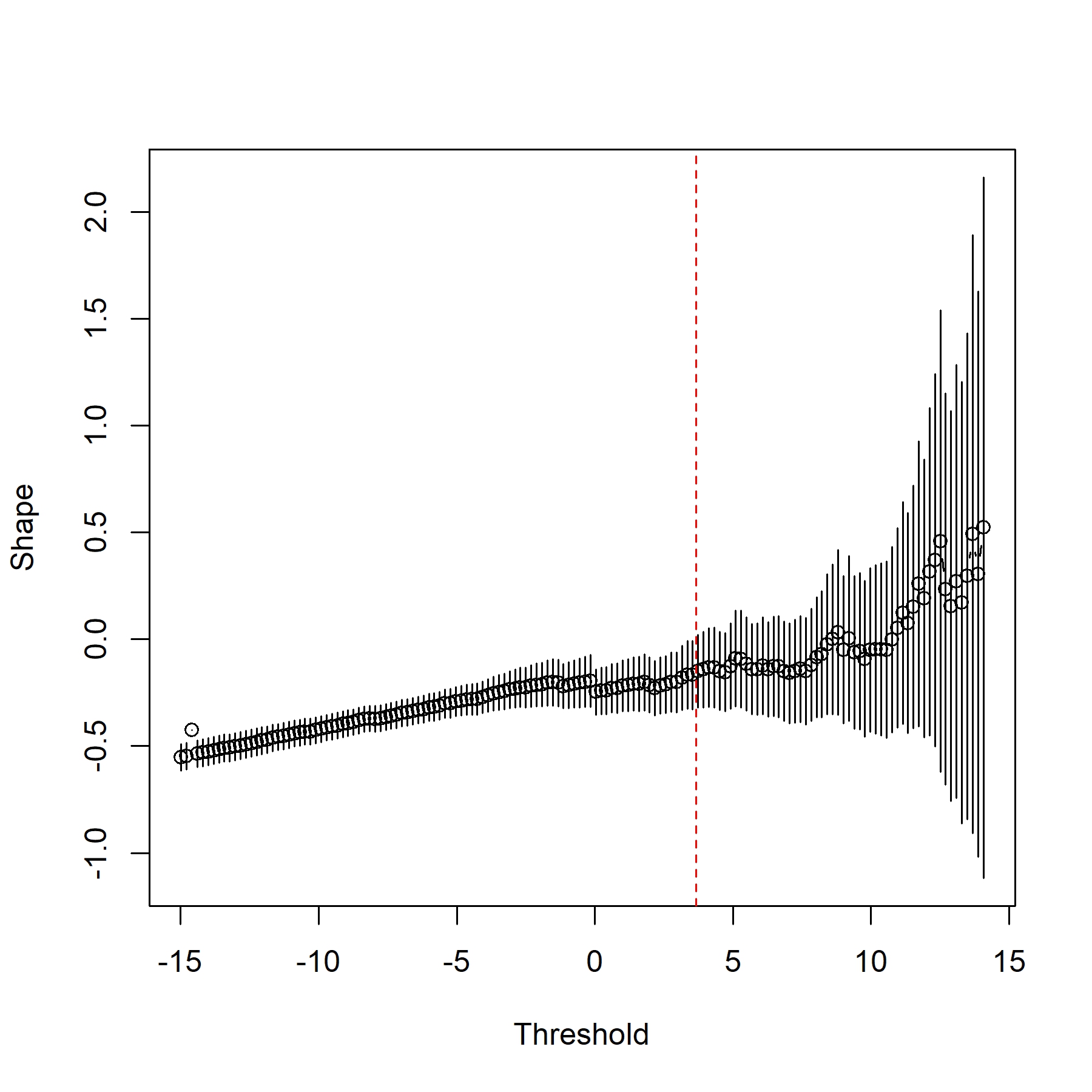

Supplement: Supplementary file 1 — Supplementary Material 1. [file 12889_2024_18785_MOESM1_ESM.zip › updated fig/stablity_shape.jpeg]

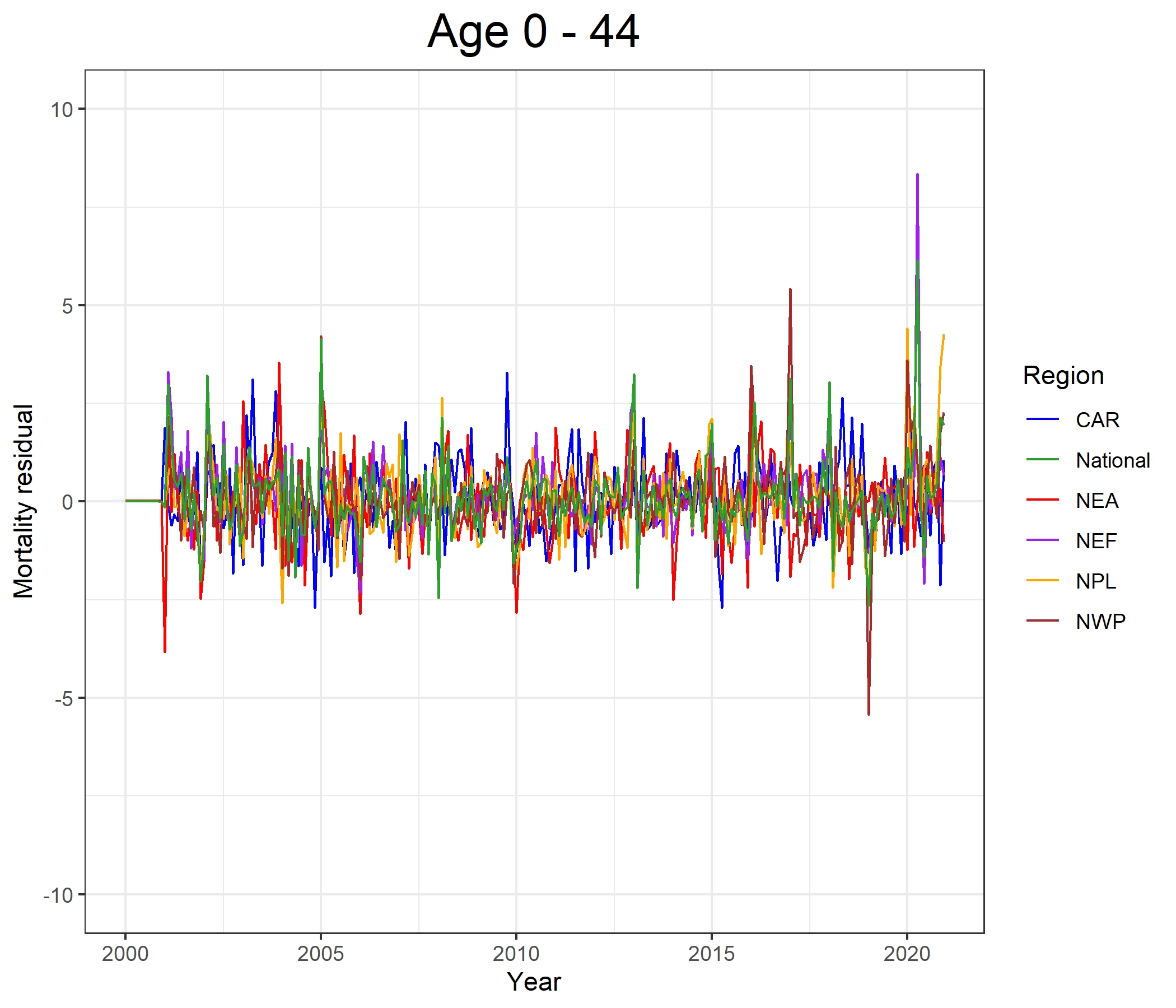

Supplement: Supplementary file 1 — Supplementary Material 1. [file 12889_2024_18785_MOESM1_ESM.zip › updated fig/std_mort_res_044.jpeg]

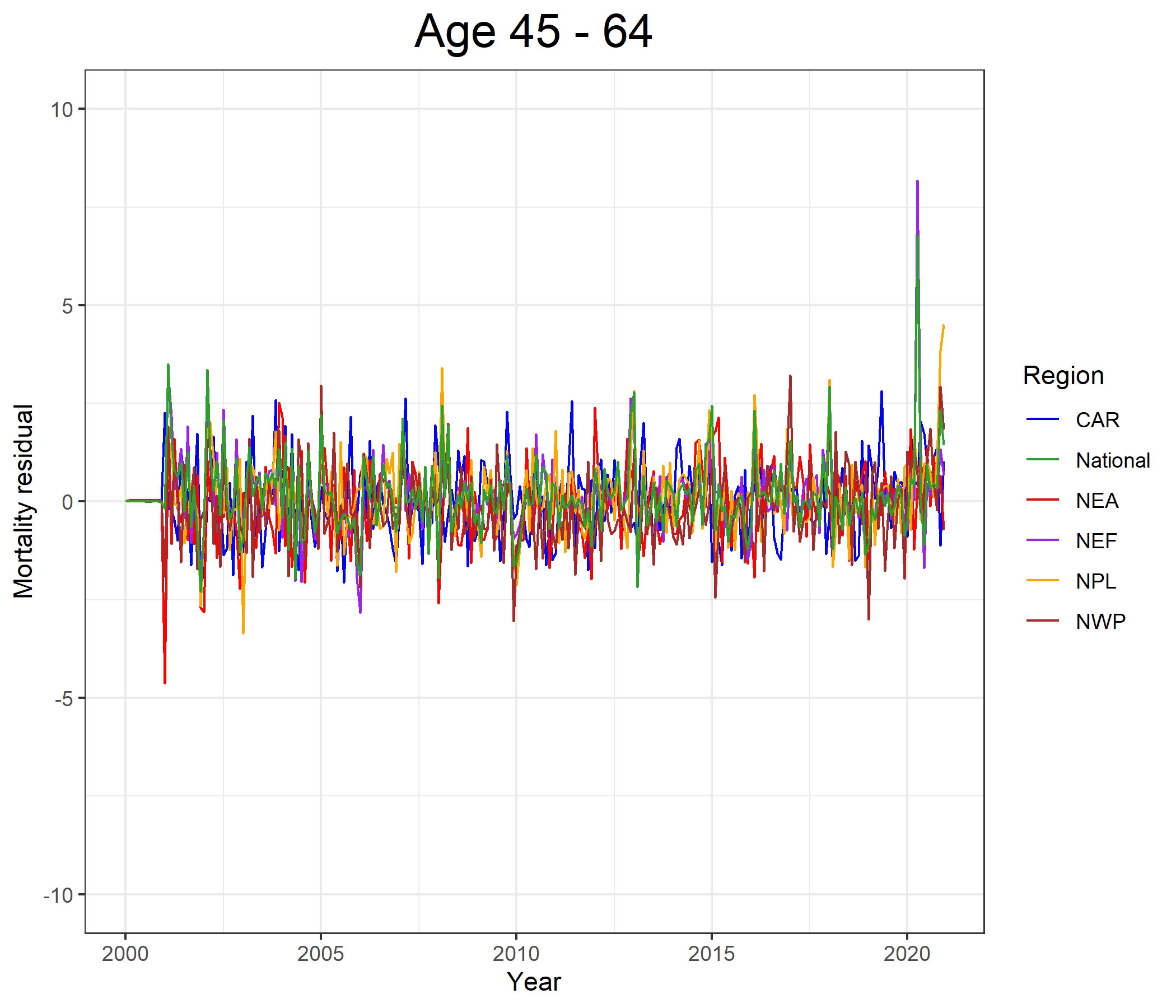

Supplement: Supplementary file 1 — Supplementary Material 1. [file 12889_2024_18785_MOESM1_ESM.zip › updated fig/std_mort_res_4564.jpeg]

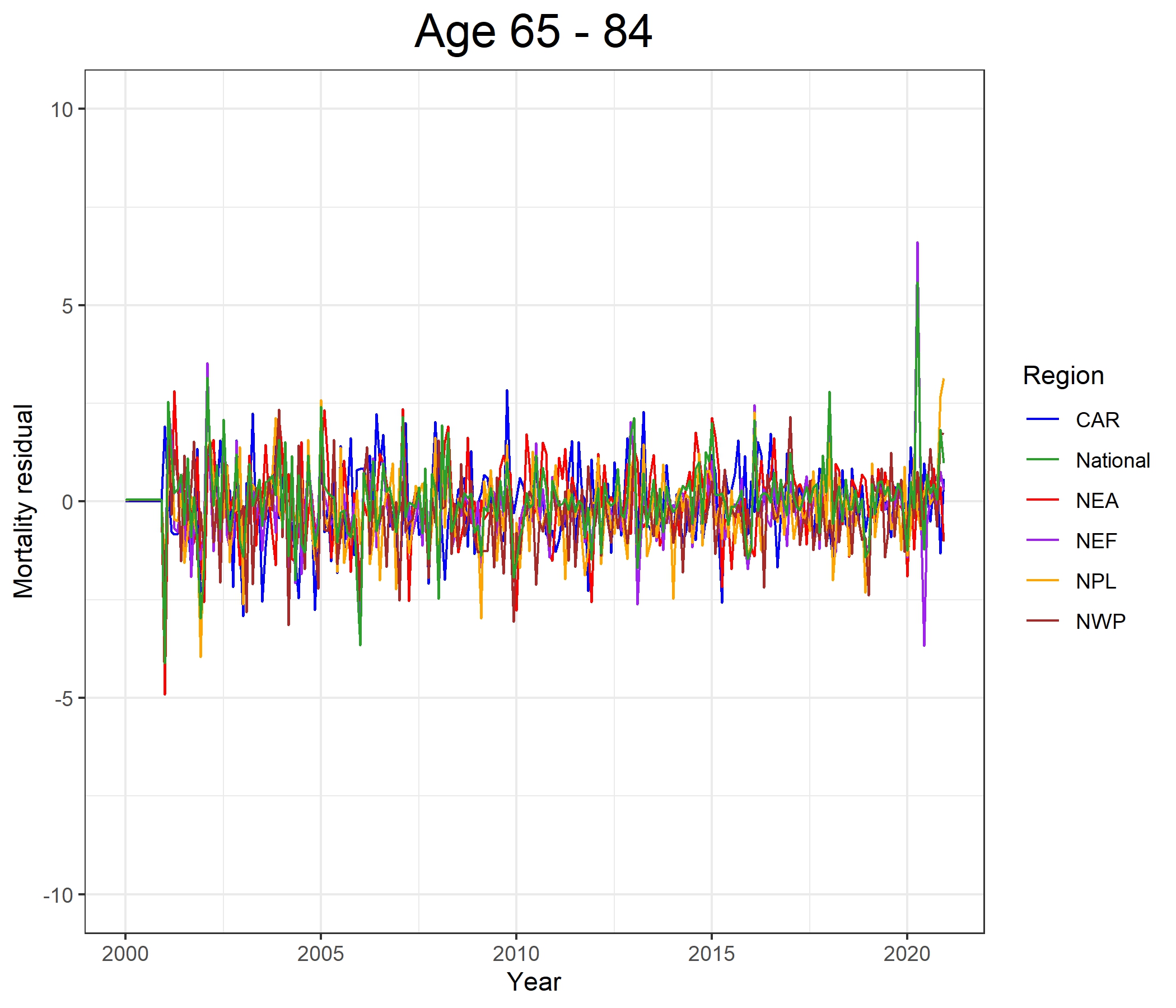

Supplement: Supplementary file 1 — Supplementary Material 1. [file 12889_2024_18785_MOESM1_ESM.zip › updated fig/std_mort_res_6584.jpeg]

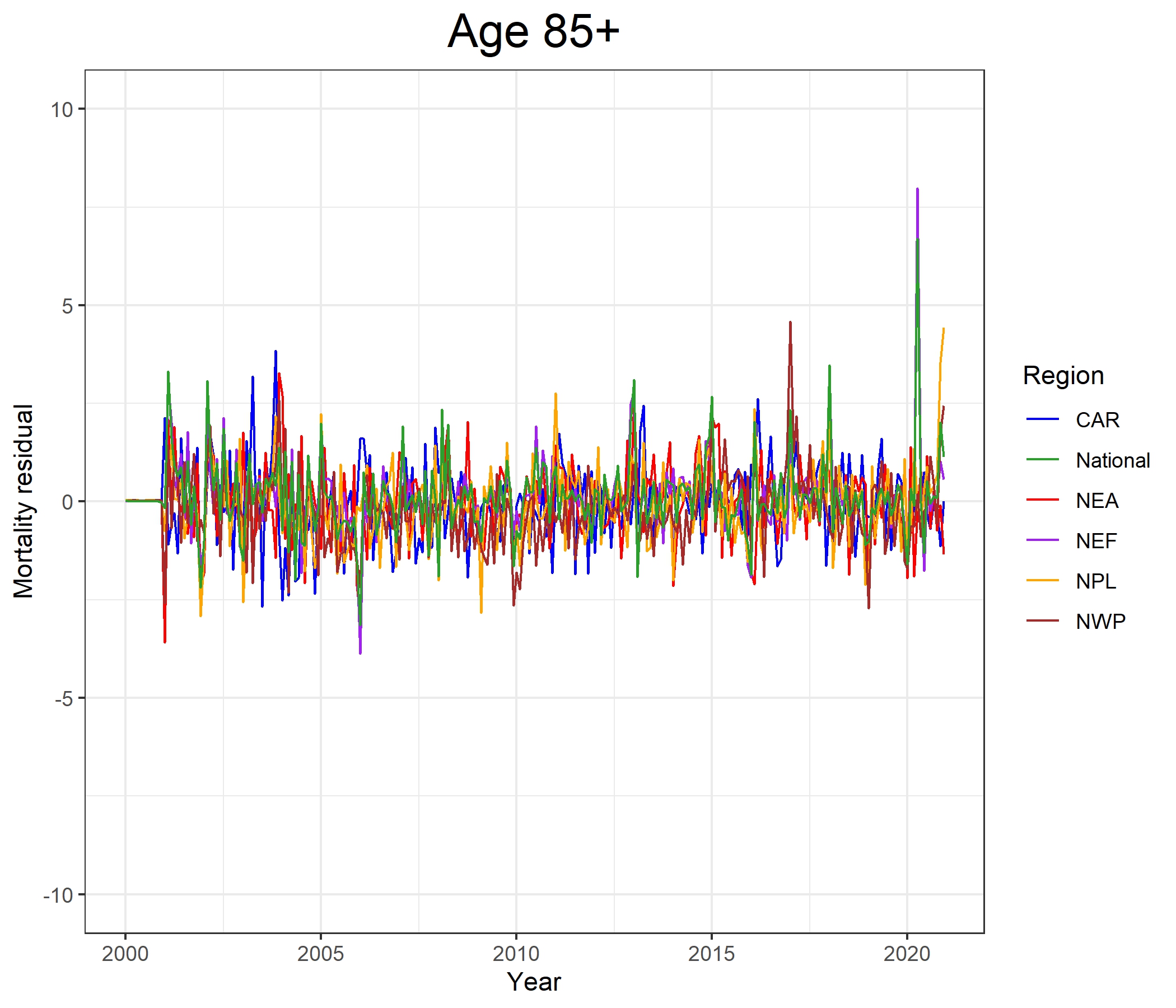

Supplement: Supplementary file 1 — Supplementary Material 1. [file 12889_2024_18785_MOESM1_ESM.zip › updated fig/std_mort_res_85.jpeg]
